# Unlocking students’ potential beyond traditional exams: the influence of collaborative testing on nursing students’ retention and soft skills

**DOI:** 10.1186/s12912-025-03237-z

**Published:** 2025-05-26

**Authors:** Amal Diab Ghanem Atalla, Mohamed Hussein Ramadan Atta, Amina Hemida Salem Ghattas

**Affiliations:** 1 Department of Nursing Administration, Faculty of Nursing, Alexandria University, Alexandria, Egypt; 2 Psychiatric and Mental Health Nursing Department, Faculty of Nursing, Alexandria University, 9 Edmond Vermont Street, Alexandria, Smouha, Alexandria Egypt; 3 Critical Care & Emergency Nursing Department, Faculty of Nursing, Alexandria University, Alexandria, Egypt

**Keywords:** Collaborative testing, Retention, Soft skills

## Abstract

**Background:**

Nursing is both an art and a science, demanding specific qualities and competencies from students to succeed. These attributes are essential for effective practice and long-term professional growth. If not developed, they may negatively impact patient outcomes and safety, contributing to medical errors, higher mortality rates, and job dissatisfaction.

**Aim:**

This study examines the impact of collaborative testing on students’ retention of emergency nursing concepts and the development of soft skills.

**Design:**

A quasi-experimental research design was employed, adhering to the STROBE guidelines.

**Methods and tools:**

A total of 100 students enrolled in an emergency nursing course participated in the study. They were randomly assigned to either a control group (50 students) or an experimental group (50 students). Data was collected using the Emergency Nursing Academic Achievement Questionnaire and the Collaborative Testing Satisfaction Survey.

**Results:**

The findings revealed a significant improvement in the intervention group’s post-lecture test scores for tests 2, 3, 4, and 5 (*p* < 0.001), while no significant difference was observed in the first post-lecture test between the two groups (*p* = 0.415). Additionally, the intervention group demonstrated significantly higher scores in both the mid-term and final written examinations, indicating enhanced retention of course material (*p* < 0.001). Furthermore, 84% of students reported high satisfaction, while 14% expressed moderate satisfaction with the collaborative testing experience. They also perceived an improvement in their soft skills, with an overall score of 95.64 ± 4.35.

**Conclusion:**

The results of this study suggest that engaging students in collaborative testing significantly enhances their retention of emergency nursing concepts compared to individual testing. Moreover, most students found the collaborative experience highly satisfactory, reporting improvements in their comprehension of course material, critical thinking, problem-solving, communication, and engagement, key competencies for nursing professionals. Additionally, students recommended implementing this approach in other courses.

**Nursing Implications:**

Incorporating collaborative testing into nursing curricula can promote deeper learning and long-term knowledge retention of critical emergency concepts. From a practical standpoint, this model simulates real-world nursing teamwork, allowing students to practice decision-making, communication, and mutual accountability. It can also help reduce test anxiety, encourage peer learning, and better prepare students for interdisciplinary collaboration and emergency response scenarios they will encounter in clinical practice. Nurse educators should consider adopting collaborative assessment strategies as part of a broader pedagogical shift toward active, team-based learning that mirrors the collaborative nature of modern healthcare settings.

**Clinical trial number:**

Not applicable.

**Supplementary Information:**

The online version contains supplementary material available at 10.1186/s12912-025-03237-z.

## Background

The nursing profession is rewarding, but it also comes with many challenges. To deal with these challenges, the students who will thrive within this profession should be equipped with specific qualities and attributes [1, 2]. Some of these attributes are called hard skills or competencies, and others are called soft/interpersonal skills or competencies [3]. Hard skills are clinical competencies that include knowledge and hands-on skills essential to performing life-saving and other nursing procedures, such as inserting an oropharyngeal airway, performing basic life support in a collapsed patient or victim, and triaging several patients who come to the emergency department after a motor vehicle accident. However, an emergency nursing career requires much more than knowledge and clinical competencies. Interpersonal or soft competencies or skills are as important as hard skills to achieve success. They supplement clinical skills and lead to improved patient care outcomes. Students practice soft skills while accomplishing academic goals. Soft skills include critical thinking, communication, decision-making, and teamwork [4–7].

### Literature review

The emergency nurse plays a constant role, filled with minute-to-minute judgments about patient care scenarios and plenty of problems that require problem-solving and decision-making capabilities [8, 9]. Critical thinking and analytical skills are essential to interpreting the patients’ data and making decisions on the spot, especially in triage situations. Additionally, many patients are admitted to the emergency department, and the nurses must be able to communicate and collaborate with healthcare teams [10, 11]. Effective, clear communication and collaboration are required to keep every healthcare member on track and operate with the same information as one another. Based on a long history of excellence in nursing education and through the adoption of teaching and assessment methods to prepare students with these attributes, the leading instructors and professors of nursing programs should promote and reinforce these top nursing qualities in all students [3, 12–16].

The preparation of nursing students to work collaboratively is one of the main interpersonal competencies [17, 18]. The Quality and Safety Education for Nurses (QSEN) guidelines consist of six competencies, one of which is teamwork and collaboration. This competency enables effective communication, commitment, respect, and sharing of the patient’s data to reach the appropriate care decision to achieve quality patient care [19]. Today, all higher educational institutions are recommending the recruitment and implementation of active learning and assessment strategies in which students work together to develop and improve their knowledge or competence on topics such as study, achievement, reasoning, etc., where they take responsibility for developing and improving these skills [20–23].

The concept of collaborative testing, also known as collaborative quizzes, where students work together to finish a test or a quiz, is generally regarded as an extension of cooperative learning. This method of assessment is based on a team approach, where students discuss the test question extensively and reach an agreement before answering it [24, 25]. In addition, active learning and assessment strategies can help students improve their academic achievement and engagement. Moreover, students have emotional and intellectual support that enables them to go beyond their current knowledge and skills to achieve common goals, improve their understanding of and ability to synthesize and integrate material, and reduce test anxiety. In the collaborative test, the students with high ability support the other students with moderate and low skills to analyze and understand the stems of the questions and to find the correct answer among the distractors, which is the essence of collaborative testing. Additionally, collaborative testing is among the methods that decrease test anxiety, as reported by the students [26–29].

### Significance of study

Improving students’ recollection of emergency nursing principles is a crucial component of nursing education, which makes this study noteworthy. Rapid decision-making, critical thinking, and a thorough comprehension of the theoretical ideas that underpin practice are all necessary for emergency nursing. By examining the effects of collaborative assessment, this study reveals a cutting-edge teaching method that improves students’ capacity to successfully retain and apply knowledge. Students who are better able to retain fundamental ideas not only perform better academically but are also more equipped to deal with emergencies in the real world with competence and confidence.

Additionally, the study highlights the value of cultivating soft skills like problem-solving, cooperation, and communication qualities that are critical for contemporary healthcare workers. By giving students, the chance to communicate meaningfully with their classmates, collaborative testing helps them develop a collaborative mindset that reflects the interdisciplinary character of healthcare organizations. The study helps to create well-rounded nursing graduates who are better able to handle challenging clinical settings and collaborate with peers to guarantee the best possible patient outcomes by encouraging these soft skills.

The potential for this research to impact nursing education methods worldwide accounts for its wider significance. Innovative teaching techniques like collaborative testing can help overcome the drawbacks of conventional assessment techniques like rote memorization and individual competitiveness as the need for highly qualified emergency nurses grows. The results of this study can encourage educators to implement evidence-based practices that emphasize skill development and information retention, which will ultimately improve nursing education quality as well as the general efficacy and safety of healthcare delivery systems. The study’s objective was to fill a knowledge vacuum in this field. Consequently, this study aims to determine the effect of implementing a collaborative test format on students’ retention of emergency nursing concepts and soft skills among those enrolled in the emergency nursing course. Also, to explore the students’ satisfaction with a collaborative test format.

### The research hypotheses are:

#### Research hypotheses

- **Primary Hypothesis**: Collaborative testing significantly improves students’ retention of emergency nursing concepts compared to individual testing methods.
- **Secondary Hypothesis**: Collaborative testing enhances the development of soft skills, such as teamwork, communication, and problem-solving among nursing students.
- **Null Hypothesis**: There is no significant difference in students’ retention of emergency nursing concepts or improvement in soft skills between those who undergo collaborative testing and those who use traditional individual testing methods.

## Methods

### Study design

A quasi-experimental design following STROBE guidelines was employed to conduct the current study.

### Setting

The study was conducted in the Critical Care and Emergency Department of the University of Alexandria’s Faculty of Nursing. The University of Alexandria’s Faculty of Nursing’s Critical Care and Emergency Department was selected as the study location because it plays a crucial part in preparing nursing students for stressful, real-world clinical situations. The influence of collaborative assessment on knowledge retention and skill development can be evaluated in this department, which specializes in teaching emergency nursing principles. The study’s goals are closely aligned with the dynamic and fast-paced nature of emergency treatment, which necessitates a thorough comprehension of theoretical concepts, prompt decision-making, and successful teamwork, all of which are important areas that collaborative testing aims to assess.

Furthermore, the department ensures that the study findings are relevant and applicable by giving access to a varied population of nursing students who are actively learning emergency nursing topics. Because of its well-established reputation for clinical training and academic brilliance, the study may be carried out in a controlled setting that encourages creative teaching strategies. The viability of adopting and assessing collaborative testing is further ensured by the educators’ and students’ familiarity with the department’s curriculum, resources, and assessment practices. By choosing this location, the study’s objectives are in line with the practical realities of nursing education, providing insightful information that can have a direct impact on critical care and emergency nursing teaching methods. The results from this situational context could influence more extensive modifications to nursing education, especially in fields that demand a high degree of application of soft skills and retention of knowledge.

### Sampling

Convenience sampling was used in this study’s participant recruitment. For the study, 100 nursing students who were enrolled in the emergency nursing course at the University of Alexandria’s Faculty of Nursing during the fall semester of the 2023–2024 academic year were chosen. Because students actively engaging in the course are accessible and available, convenience sampling was selected to ensure a quick and effective data-gathering approach. Criteria for Inclusion:

Students who, during the designated semester, were formally enrolled in the emergency nursing course. Participants in the study were students who gave their informed consent.

Students who participated in all mandatory emergency nursing course sessions. Criteria for Exclusion: Students who declined to take part in the research. Students with previous emergencies or critical care nursing clinical or professional experience may have an impact on the study’s findings.

Students who missed over 20% of the semester’s class sessions. To compare the pre-test and post-test scores of 100 students using paired t-tests, G*Power calculated the actual power for this sample size, showing whether 100 students (50 per group) is sufficient to detect a medium-sized effect with 80% power at a 5% significance level as follows:

- Test Type: Paired t-test (for pre-test vs. post-test).
- Effect Size (Cohen’s d): Medium (0.5).
- Alpha: 0.05 (5% significance level).
- Power: 0.80 (80% power).
- Sample Size (N): 100 students.

### Instruments

Tools: Two tools were used to collect data for the current study.

#### Tool one: Emergency nursing academic achievement questionnaire (ENAAQ) (supplementary file)

The questionnaire used in this study was not newly developed but was adopted by a validated and reliable emergency nursing question bank. This question bank was originally developed to fulfill the requirements of the National Accreditation Commission for Education in Nursing (NACEN), which has accredited the Faculty of Nursing at the University of Alexandria twice. Its development involved a rigorous, multi-phase process conducted by a panel of content experts to ensure the authenticity and accuracy of the knowledge assessed.

The construction of the question bank involved multiple stakeholders, including course coordinators who contributed insights aligned with the actual learning experiences provided, medical educators who ensured technical accuracy in item formulation, and specialized personnel from the Assessment and Evaluation Unit at the University of Alexandria who oversaw psychometric evaluations. Items in the bank were continuously piloted by students, and their performance was systematically reviewed through item analysis to determine difficulty and discriminatory indices. These analyses were conducted both before and after administration to validate item quality. Based on this process and expert feedback, items were either retained, revised, or suspended from use until corrections were made.

Furthermore, the language and clarity of the items were carefully reviewed and revised to ensure comprehensibility and consistency. Recognizing the dynamic nature of the medical field, the question bank undergoes regular re-evaluation every 3–5 years to incorporate new knowledge and ensure ongoing content and construct validity.

For the current study, the researcher carefully selected relevant items from this question bank to align with the study objectives, ensuring that the content was both valid and reliable for assessing emergency nursing knowledge. Due to the comprehensive and validated nature of the question bank, we are confident in the appropriateness and psychometric robustness of the questionnaire used in our study. The Emergency Nursing Academic Achievement Questionnaire comprises three parts:

### Part I: Demographic and academic data of the participating students

This part was developed by the researcher to collect the sociodemographic and academic data of the participants, including age, sex, and grade point average (GPA).

### Part II: Formative post-lecture tests (supplementary file)

Five post-lecture tests, 10 multiple–choice questions each, were selected manually from the MCQs pool by the researcher (course coordinator) from the validated, reliable emergency nursing questions bank. Based on a blueprint and submitted to 3 professors in the specialty to review and ensure that all items were in line with the overall objectives of each lecture, before providing for both groups.

### Part III: Summative mid–semester & final written exams (supplementary file)

A midterm of 40 multiple–choice items and a final examination of 70 questions were extracted from the question bank. To check the face and content validity of the exams, they were submitted to five experts, 2 in the exam construction and three professors in the field of specialty. The suggested modifications were made accordingly. The control and experimental groups were exposed to the same exams, and they finished the exams individually. Both groups’ performance in the post-lecture tests, mid-semester, and final written exams were scored according to the Egyptian Academic Setting’s Operational Scoring System (Excellent: 85-100%, Very good: 75% < 85%, Good: 65%-< 75%, Pass: 60%-< 65%, and Fail: < 60%.).

### Tool two: Collaborative testing satisfaction survey (supplementary file)

Before implementing the collaborative testing satisfaction survey in the present study, it was first created and tested on students enrolled in the critical care course in the semester before the current study. The survey development process adhered to the recommended steps for creating questionnaires and surveys. Once the study’s objectives were established, a literature review focusing on objectives similar to the current study was conducted [30–34]. A survey of 30 items was conducted.

### Tools validity

To establish face validity and effectively capture the topic under investigation, a survey was submitted to five experts in emergency nursing and nursing education departments, and the recommended modifications were made accordingly (unclear, double-barreled, and confusing statements were omitted). Three items were omitted from the survey (27 items).

To confirm correctness, a confirmatory factor analysis was conducted for tool one, the Emergency Nursing Academic Achievement Questionnaire (ENAAQ), and tool two, the Collaborative Testing Satisfaction Survey. The Kaiser-Meyer-Olkin (KMO) and the Bartlett Test of Sphericity were the first instruments used to evaluate sample adequacy, which was ensured in some settings. For Bartlett’s Test of Sphericity, a minimum KMO value of 0.60 and a significance level of 0.05 are required.

The results showed that the Collaborative Testing Satisfaction Survey scale had a value of 0.921 (P 0.000), and the Emergency Nursing Academic Achievement Questionnaire (ENAAQ) had a value of 0.928 (P 0.000). The fact that factor loadings for every idea in this study were higher than the recommended cutoff of 0.70. backed up the scales’ construct validity. The average variance extracted (AVE) values for each study variable dimension are also provided. Show that convergent validity is satisfied. Convergent validity was assessed using the average variance extracted (AVE) values for every component. AVE values greater than 0.50, which demonstrate that the construction explains most of the variance, are indicative of convergent validity. Discriminant validity was assessed by comparing the squared correlations between the constructs with the AVE values. Discriminant validity was considered met as each AVE value was greater than the squared correlations. Consequently, it was discovered that the measures used in this study had both discriminant and convergent validity.

### Ethical considerations

The Research Ethics Committee of the Alexandria University Faculty of Nursing gave its approval to the study protocol. Students were informed of the study’s objectives before their consent. Every questionnaire was given a code number to guarantee privacy and identity protection. Students were told that the data would only be used for research. The possibility of dropping out of the study has been verified.

### Pilot study and reliability

The survey was piloted on 15 students who were registered for a critical care nursing course after their exposure to 3 collaborative testing experiences. A survey was distributed to the students immediately after exposure to collaborative experiences, and it was redistributed again by the end of the semester (92 students, 4 students were absent). Then all surveys (first and second students’ responses) were entered into a spreadsheet and submitted to the statistics to assess underlying components using principal components analysis (PCA) (± 0.78) and the internal consistency of the survey (Cronbach’s alpha coefficient 0.94). The analysis revealed that the five items were irrelevant and were dropped.

The final form of the survey consists of 22 items distributed along with 4 themes: critical thinking & decision-making (7 items), communication & teamwork (7 items), academic motivation & accountability & engagement (4 items), self-confidence & self-esteem (3 items), and overall item that indicates if the students would recommend this assessment method for other courses. in addition to one open end question asking the students about the drawbacks of the collaborative quiz technique. The total score ranged between (22–110), the higher score reflects a high satisfaction level, and the subscales include: 22 less than 55 = had a low satisfaction, 55 less than 82.5 had moderate satisfaction, and 82.5–100 had high satisfaction.

A valid and reliable self-administered 5-point Likert scale (5 = strongly agree, 4 = agree, 3 = neutral, 2 = disagree, and 1 = strongly disagree) opinionnaire was used to explore perceptions and satisfaction with the collaborative testing experience.

### Overcame the problem of common method biases

To combat the issue of common method bias (CMB), the researchers employed a mix of design, procedural, and statistical controls. Participants’ identity and confidentiality are formally guaranteed to reduce social desirability bias and encourage honest responses. They also separated the different metrics inside the questionnaire, providing alternate response forms and clear instructions to lessen the likelihood that respondents would provide the same answers on numerous items. Using Harman’s single-factor test, the author’s statistical analysis showed that no single factor explained the bulk of the variance, indicating that CMB was not a serious worry.

Confirmatory factor analysis (CFA) was also used to evaluate the measurement model and ensure that the structures were distinct and not overly linked, which helped to lower the likelihood of CMB. The authors also conducted a preliminary survey to refine the questions, guarantee clarity, and eliminate any ambiguity that might result in CMB. By combining these techniques, the authors were able to successfully lessen the potential impact of common method bias on the results of their investigation.

### Data collection

Before collecting data, the researcher explained the purpose and procedures of the study to the students, including that confidentiality was assured and participation in the study was voluntary. Obtained informed consent from all students; the students had the right to withdraw from the study at any time. The information gathered was kept confidential. No personal details of participants were collected, which compromised anonymity. During the first lecture with the students who registered for the Emergency Nursing Course, the researcher (instructor) welcomed the students and briefed them about the purpose of the research. The students were stratified by their grade point average (GPA) in the last year and distributed into two equal groups: control group (*n* = 50 students: those who were engaged in traditional (individual-based - test) and experiment group (*n* = 50 students: those who were engaged in a collaborative-based test format to establish homogeneity between both groups.

The experimental group was stratified again by their grade point average (GPA) and divided into 10 equal subgroups (5 students each), numbered groups 1–10, and these groups were maintained for the entire semester. During the semester, both groups had three hours of lecture per week with an overall length of 36 h. The teacher, the teaching methods, the course material, and the test content were identical for the two groups. Throughout the semester, students in both groups were exposed to 7 examinations (5 post-lecture tests, one midterm, and one final comprehensive exam). These exams were extracted by the instructor from the questions bank of the course. Ten minutes were provided for the control group to complete the 10 MCQ questions. An additional 10 min were provided to the experimental group to discuss the questions and complete the test collaboratively. Each group attended the exam in a separate class. Both groups were exposed to the same midterm and comprehensive final tests using the individual-based format.

To determine the effect of implementing the collaborative test on the students’ academic achievement, the overall scores of all tests completed by the collaborative group were compared with the overall scores of all tests completed by the individual group. In both groups, the number of students who passed and did not pass was calculated. In the overall course grade, a passing score of 60% or more. After completing the final comprehensive test, the experimental group of students was asked to complete a self-administered, 5-point Likert scale to rate their satisfaction with the collaborative test.

### Data analysis

The IBM Statistical Package for the Social Sciences (SPSS) software package version 23.0 was used to feed the data to the computer and analyze it. The Chi Monte Square test was used to assess the comparison of groups according to specific variables. Two categories of generally distributed quantitative variables were compared using a student’s test. The significance of the results obtained was considered at a level of 5%.

## Results

Table 1 shows that the mean age scores in both groups were not statistically significant (*p* = 0.181). Female students were dominant in both groups, with no statistical significance (*p* = 0.817). A similar result was observed with the variable of entry-level to the program. Finally, the first-year grade point average (GPA) did not differ significantly (MCp = 1.000), indicating homogeneity between the control and experimental groups.

**Table 1:** Distribution of the studied groups according to demographic and academic characteristics

Variables	Experimental (*n* = 50)		Control (*n* = 50)		χ^2^/t	*P*
	No	%	No	%		
**Age**						
19-<21	17	34.0%	21	42.0%	χ^2^ = 1.760	0.415
21-<23	21	42.0%	22	44.0%		
≥ 23	12	24.0%	7	14.0%		
Mean ± SD	21.28 ± 1.28		20.96 ± 1.09		t = 1.348	0.181
**Gender**						
Male	12	24.0%	13	26.0%	χ^2^ = 0.053	0.817
Female	38	76.0%	37	74.0%		
**Level of entry to the faculty**						
Secondary school	38	76.0%	37	74.0%	χ^2^ = 0.053	0.817
Technical institute	12	24.0%	13	26.0%		
**Grade point average (GPA)**						
2-<2.5	1	2.0%	1	2.0%	χ^2^ = 0.066	^MC^*p*= 1.000
2.5-<3	11	22.0%	11	22.0%		
3-<3.5	27	54.0%	28	56.0%		
≥ 3	11	22.0%	10	20.0%		
Mean ± SD	3.76 ± 4.38		3.14 ± 0.35		t = 0.988	0.325

χ^2^: Chi-square test MC: Monte Carlo t: Student t-test

Table 2 shows that to determine the effect of collaborative testing on undergraduate students’ retention of emergency nursing concepts. 100 students were recruited and divided into two groups: the control group (*n* = 50) receiving individual-based tests and the experimental group (*n* = 50) receiving collaborative testing. The scores of the tests in both groups were compared. It can be noted that there was a significant difference between the average scores of the 2nd, 3rd, 4th, and 5th post-lecture tests among the control and experimental groups (*p* < 0.001). However, there was no significant difference between the two groups regarding the 1st post-lecture test (*p* = 0.004). Similar significant trends were reflected in the mid-semester and final tests of both groups (*p* < 0.001 & *p* < 0.001) respectively.

**Table 2:** Analysis of variance results for grades of the two studied groups in emergency nursing course

Quiz #	Alpha Grades	Experimental (*n* = 50)		Control (*n* = 50)		χ^2^/t	*p*
		No	%	No	%		
First Quiz	Fail	40	80	46	92	**χ**^**2**^ **=** 2.99	0.084
	Pass	10	20	4	8		
	**Mean ± SD**	4.50 ± 1.04		3.84 ± 1.18		t = 2.967^*^	0.004^*^
Second Quiz	Fail	0	0	18	36.0	**χ**^**2**^ **=** 61.275^*^	^MC^*p*<0.001^*^
	Pass	0	0	16	32.0		
	Good	10	20	11	22.0		
	V. good	25	50	3	6.0		
	Excellent	15	30	2	4.0		
	**Mean ± SD**	8.10 ± 0.71		5.98 ± 1.27		t = 10.316^*^	< 0.001^*^
Third Quiz	Fail	0	0	9	18.0	**χ**^**2**^ **=** 61.464^*^	< 0.001^*^
	Pass	0	0	15	30.0		
	Good	5	10	19	38.0		
	V. good	30	60	7	14.0		
	Excellent	15	30	0	0.0		
	**Mean ± SD**	8.30 ± 0.79		6.48 ± 0.95		t = 10.404^*^	< 0.001^*^
Fourth Quiz	Fail	0	0	10	20.0	**χ**^**2**^ **=** 66.92^*^	^MC^*p*<0.001^*^
	Pass	0	0	19	38.0		
	Good	5	10	16	32.0		
	V. good	20	40	3	6.0		
	Excellent	25	50	2	4.0		
	**Mean ± SD**	8.50 ± 0.81		6.34 ± 1.04		t = 11.548^*^	< 0.001^*^
Fifth Quiz	Fail	0	0	18	36.0	**χ**^**2**^ **=** 63.598^*^	< 0.001^*^
	Pass	0	0	15	30.0		
	Good	10	20	13	26.0		
	V. good	25	50	4	8.0		
	Excellent	15	30	0	0.0		
	**Mean ± SD**	8.10 ± 0.71		5.94 ± 1.17		t = 11.188^*^	< 0.001^*^
Mid-Semester Exam	Fail	0	0	1	2.0	**χ**^**2**^ **=** 47.574^*^	^MC^*p*<0.001^*^
	Pass	0	0	2	4.0		
	Good	0	0	23	46.0		
	V. good	23	46	22	44.0		
	Excellent	27	54	2	4.0		
	**Mean ± SD**	34.32 ± 2.24		29.12 ± 2.85		t = 10.125^*^	< 0.001^*^
Final Exam	Fail	0	0	2	4.0	**χ**^**2**^ **=** 24.614^*^	^MC^*p*<0.001^*^
	Pass	0	0	10	20.0		
	Good	14	28	21	42.0		
	V. good	18	36	14	28.0		
	Excellent	18	36	3	6.0		
	**Mean ± SD**	56.84 ± 5.79		50.16 ± 5.64		t = 5.842^*^	< 0.001^*^

χ^2^: Chi-square test MC: Monte Carlo

t: Student t-test *: Statistically significant at *p* ≤ 0.05

Table 3 reveals the level of satisfaction of the experimental group with the collaborative testing. To explore the effect of collaborative testing on the students’ soft skills and their level of satisfaction with the collaborative testing experience, the mean percent score of more than three-quarters of the students agreed and strongly agreed that the collaborative test experience had improved their critical thinking & decision making (79.93 ± 8.25). Moreover, the majority of the students agreed and strongly agreed that their communication & teamwork skills improved when they engaged in the collaborative test experience (82.57 ± 8.83). Among the four soft skills, academic motivation & accountability & engagement were agreed by the highest percent scores of the students (90.25 ± 5.53) as they improved through the collaborative testing experience.

**Table 3:** Level of satisfaction of the experimental group with the collaborative testing

Collaborative quizzes survey scales	(*n* = 50)	
	Total score	Mean percent score
− Critical thinking & decision making	29.38 ± 2.31	79.93 ± 8.25
− Communication & teamwork	30.12 ± 2.47	82.57 ± 8.83
− Academic motivation & accountability & engagement	18.44 ± 0.88	90.25 ± 5.53
− Self-confidence & self-esteem & test anxiety	17.70 ± 1.80	85.63 ± 11.24
**Overall Score**	**95.64 ± 4.35**	**83.68 ± 4.94**
**Overall Levels of Satisfaction**	**No.**	**%**
− Moderate satisfaction	8	16.0
− High satisfaction	42	84.0

**Fig. 1:**
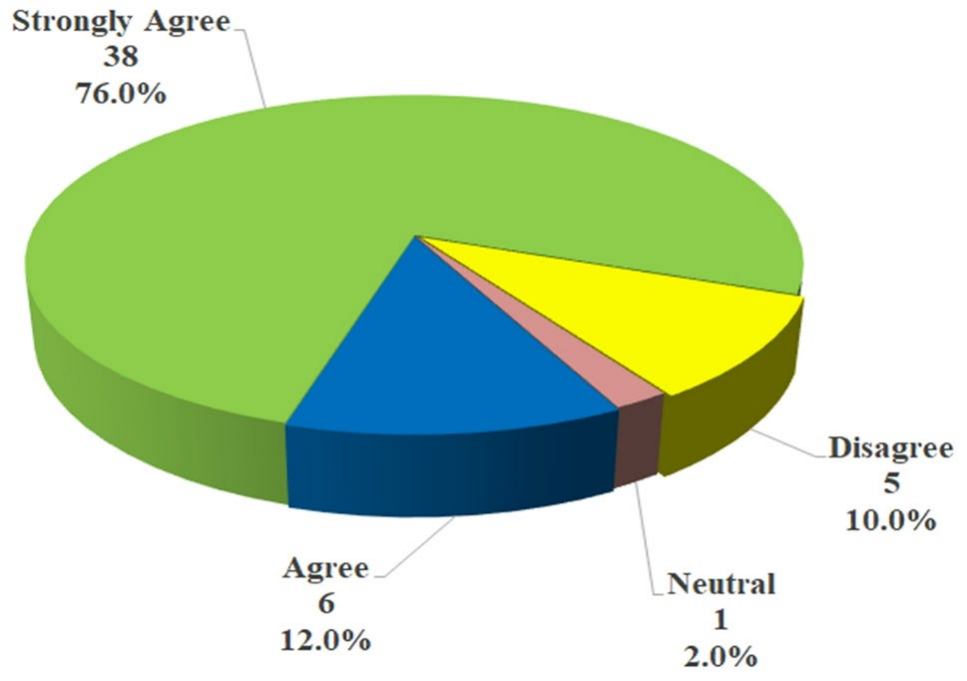
Overall experimental group perception toward the collaborative testing experience

Additionally, self-confidence & self-esteem & test anxiety were improved as mentioned by 85.63 ± 11.24 of the students. Furthermore, the vast majority of the students (84%) were highly satisfied with the collaborative tests’ method, compared to 16% who were moderately satisfied. Additionally, an analysis of the overall statement [22] in the survey dedicated to determining the extent of how the experimental group recommended the adoption of collaborative testing in other courses, Fig. 1 displays that the majority of the students strongly agreed and agreed (76% & 12% respectively) to adopt this experience in other courses, 2% did not know (neutral), while 10% of the students did not agree.

## Discussion

### Primary hypothesis

Collaborative testing significantly improves students’ retention of emergency nursing concepts compared to individual testing methods.

The hypothesis that students who participate in collaborative testing retain emergency nursing concepts better than those who do not was effectively proven and supported by the current study. This can be evidenced by a significant improvement in the grades of students who participated in collaborative-based format testing in their 2nd, 3rd, 4th, and 5th post-lecture tests, as well as the midterm and final written examinations. This improvement can be attributed to several factors. In the collaborative testing, each group included both high-ability (high GPAs) and low-ability (low GPAs) students, facilitating in-depth discussions, explanations of course materials, and analysis of questions, leading to better consensus and strategies for answering questions, resulting in improved grades. Additionally, high-ability students helped their peers understand the course content, analyse questions, and provide psychological support. Furthermore, the researcher observed a more relaxed test atmosphere and lower test anxiety, potentially due to the absence of a proctor’s role. Cheating was less of a concern during these tests, so the instructor was no longer considered the proctor. Conversely, there were no significant improvement was noted in either the control or experimental groups during the first post-lecture test. This can be attributed to the unfamiliarity of the students with a collaborative test approach, as well as a collaborative learning experience preceding the collaborative testing strategy. Furthermore, the discussions and debates took longer than expected for the students to fully grasp the process, leading to increased cohesion.

The findings are directly in line with previous findings. A similar conclusion was reached by Beniss and Tajalli (2022) in Iran. They found that there is a significant difference between the collaborative and individual groups’ performance in course achievement, in such a way that the collaborative group outperformed the individual group [4]. Moreover, the findings are directly in line with a study conducted by Efu (2019). She reviewed and analyzed 16 empirical studies from various disciplines and investigated the extent to which collaborative assessments improve student learning. Out of the 16 studies, nine found that collaborative assessments improved student learning, while seven found no difference in learning between students who completed their exams individually versus in groups [33]. Furthermore, Rivaz et al. (2015) studied 84 students enrolled in the Medical-Surgical course to assess their retention of the course material. The results indicated better test scores and continued enhancement in learning as a result of collaborative testing, compared to the traditional approach [34].

The findings indicated a significant improvement in students’ achievement due to the collaborative testing method. The researchers suggested that instructors should consider incorporating the collaborative testing approach for both learning and assessment purposes [35]. On the same line, Radford and Blount (2023) conducted a study on the impact of collaborative testing on the teamwork and collaboration of nursing students. They found that collaborative tests contributed to the enhancement of the students’ knowledge, and better comprehension of the material, and promoted deeper learning by addressing knowledge deficiencies [36]. A similar result was reported by Roger et al. (2021). They explored how collaborative testing affects nursing student content retention and student views of the collaborative testing process. Their findings suggest that implementing collaborative testing in nursing programs could enhance content retention, encourage shared decision-making among students, and lead to better patient care [16].

Contrary to the findings of the current study, Caboral Stevens and Fox (2020) compared the test scores of nursing students who completed collaborative tests with those who completed individual tests. Their results indicated that the mean knowledge retention score of the students in the collaborative test group was significantly lower. Stevens attributed these results to the long period (eight months) before the retention test was conducted. According to Stevens, students tend to forget most of the content they learned, especially when they do not use it frequently [12].

### Secondary hypothesis

Collaborative testing enhances the development of soft skills such as teamwork, communication, and problem-solving among nursing students.

The current study’s findings positively supported the hypothesis that Students who completed collaborative testing exhibited improvement in soft skills than those who did not. This can be revealed by the overall score of more than three-quarters of students (83.68 ± 4.94) strongly agreed and agreed that participation in the collaborative testing experience improved their soft skills, including critical thinking & decision-making, communication & teamwork, academic motivation & accountability & engagement, and self-confidence & self-esteem & test anxiety. In addition, 84% of the students demonstrated high satisfaction with the collaborative testing experience, and 76% recommended this experience in other courses. Students’ satisfaction with CT could be attributed to that, according to feedback from the students, collaborative tests provided them with the chance to work together with their peers and more time to analyze the exam questions, and empowered them to discuss the questions without embarrassment. The students demonstrated a feeling of satisfaction and more confidence following the collaborative tests (Olvera-Lobo et al., 2007) [37]. Similarly, Roger et al. revealed that implementing collaborative testing in nursing programs could enhance content retention, encourage shared decision-making among students, and lead to better patient care [16].

In another study conducted by Connelly (2022), he found that the students were highly motivated by the collaborative test aspect of the course and suggested an extension of this approach to other classes [38]. Furthermore, Bovee (2016) indicated a decrease in test anxiety among students who took tests collaboratively, in comparison to those who took tests individually. Bovee also found other positive outcomes such as improved test scores, increased confidence, development of critical thinking skills, and higher student satisfaction [24]. Additionally, a study that was conducted by Levy et al. (2018) showed that collaborative testing was generally considered more beneficial for learning and less stressful than traditional examinations [39].

Contrary to the result of the current study, some studies highlighted that students were not relaxed and disliked the collaborative method. These feelings may be due to some reasons, including noise and limited time in the collaborative test, and may also be due to personal attributes of the students [40, 41]. Studies conducted by Eastwood, Helmke, Zhang, Mahoney, and Wiggs revealed that students were displeased and were not relaxed with the interpersonal conflicts that usually occurred during collaboration. The negative perceptions of students towards the collaborative test strategy are linked to conflicts with groupmates. Therefore, effective conflict resolution mechanisms are necessary to ensure that collaborative testing is beneficial for both learners and instructors. [42–44].

## Conclusions

The current study’s findings indicated that students’ retention of emergency nursing concepts is significantly improved when students engaged in a collaborative-based test format compared to an individual-based test. In addition, the majority of students who engaged in the collaborative tests were highly satisfied with the collaborative experience and perceived that it improved their understanding of course material, critical thinking, problem-solving, communication, and engagement, which are critical for future nursing professionals. Additionally, they recommended that this experience be implemented on the other courses.

### Recommendations for nursing education

Nursing education must advance beyond traditional examination methods to better assess students’ clinical reasoning, teamwork, and communication skills. This study demonstrates that collaborative testing can serve as an effective educational strategy that not only enhances content retention but also fosters essential soft skills required in clinical practice. To translate these findings into educational practice, nurse educators should receive appropriate training to design and facilitate collaborative assessments that are aligned with learning objectives. Collaborative tests should be shorter than traditional individual-based exams, and group sizes should be kept small to ensure active participation from all students. Clear instructions must be provided to help students understand the transition between individual and group phases of testing. Incorporating post-test debriefing sessions can reinforce key concepts and allow students to reflect on their learning experience. Expanding the use of collaborative testing across various nursing subjects can further promote communication, decision-making, and critical thinking competencies. Faculty can also use these experiences to model professional collaboration and foster stronger educator-student relationships. While student satisfaction should not be the sole driver of educational innovation, their positive perceptions provide valuable insights for enhancing learner-centred approaches.

### Strengths & limitations

The strengths of this study include its quasi-experimental design, the use of both achievement and perception measures, and its focus on emergency nursing, a field where rapid decision-making and teamwork are essential. However, the study is limited by its single-site setting, relatively small sample size, and lack of long-term follow-up to evaluate sustained retention. Additionally, instructor influence and student familiarity with collaborative methods may have impacted results. Future research should explore the scalability of this method in diverse educational and clinical contexts.

## Electronic supplementary material

Below is the link to the electronic supplementary material.

Supplementary Material 1

Supplementary Material 2

Supplementary Material 3

Supplementary Material 4

Supplementary Material 5

Supplementary Material 6

Supplementary Material 7

Supplementary Material 8

## Data Availability

Data are available on request from the corresponding author.

## Abbreviations

QSEN: Quality and Safety Education for Nurses
ENAAQ: Emergency Nursing Academic Achievement Questionnaire
GPA: Grade Point Average
CGT: Collaborative Group Testing
CT: Collaborative Testing

## References

[1] El-Ashry AM, Hussein Ramadan Atta M, Alsenany SA, Farghaly Abdelaliem SM, Abdelwahab Khedr M. The effect of distress tolerance training on problematic internet use and psychological wellbeing among faculty nursing students: A randomized control trial. Psychol Res Behav Manage. 2023;4015. 10.2147/PRBM.S42319. 10.2147/PRBM.S423194 PMC10544047 37790728

[2] Atta M, Hammad HA, Elzohairy NW. The role of empathy in the relationship between emotional support and caring behavior towards patients among intern nursing students. BMC Nurs. 2024;23(1):443. 10.1186/s12912-024-02074-w. PMID: 38943109; PMCID: PMC11212155. 38943109 10.1186/s12912-024-02074-w PMC11212155

[3] Amin SM, El-Sayed AAI, Alsenany SA, Atta MHR, Morsy OMI, Asal MGR. (2025). How Clinical Reasoning and Decision-Making Competences Influence the Provision of Empathic Care Among Nursing Students? Teaching and Learning in Nursing. 10.1016/j.teln.2025.01.005

[4] Sadeghi Beniss AR, Tajalli F. The impact of collaborative testing on Iranian EFL learners’ course achievement. Int J Linguist Transl Stud. 2022;3(4):42–51.

[5] Eastridge JA, Benson WL. Comparing two models of collaborative testing for teaching statistics. Teach Psychol. 2020;47(1):68–73.

[6] Kuchta JL. Undergraduate Nursing Faculty Perceptions of Collaborative Testing A Dissertation. 2020.

[7] Trisyani Y, Emaliyawati E, Prawesti A, Mirwanti R, Mediani HS. Emergency nurses’ competency in the emergency department context: A qualitative study. Open Access Emerg Med. 2023;15(April):165–75. 37197564 10.2147/OAEM.S405923 PMC10183472

[8] Zoromba MA, El-Gazar HE, Noaman Malek MG, El-Sayed MM, Atta R, M. H., Amin SM. Career growth as a mediator between scope of practice, importance of practice and emergency nursing competency among school nurses. J Adv Nurs. 10.1111/jan.16783 10.1111/jan.16783 39894453

[9] Atta MH, Elsayed SM, El-Gazar HE, Abdelhafez E, N. G., Zoromba MA. Role of violence exposure on altruistic behavior and grit among emergency nurses in rural hospitals. Int Nurs Rev. 2025;72(1):e13086. 10.1111/inr.13086. 39752364 10.1111/inr.13086

[10] Ali HI, Othman AA, Atta MHR, Mohamed SS, Mohamed SH. Revelation of the mediation role of moral sensitivity on safety attitude and personality traits among critical care nurses. BMC Nurs. 2025;24:261. 10.1186/s12912-025-02868-6. 40057726 10.1186/s12912-025-02868-6 PMC11889890

[11] Elsaeed BIK, Atta MHR, Fouda ME, Ahmed HAE, El Demerdash D, Elzlbany GAM. Effect of implementing training programme for nurses about care bundle on prevention of ventilator-associated pneumonia among newborns. Nurs Crit Care. 2025;30(2):e70000. 10.1111/nicc.70000. PMID: 40074557. 10.1111/nicc.70000 40074557

[12] Caboral-Stevens M, Fox DP. The Use of Collaborative Testing with Baccalaureate Nursing Students. Teach Learn Nurs [Internet]. 2020;15(1):37–41. Available from: 10.1016/j.teln.2019.09.007

[13] Eastridge JA. Relationship Between Use of Collaborative Testing and Nursing Student Success. 2018.

[14] Eastwood JL, Kleinberg KA, Rodenbaugh DW, Phillips TA, Munn AC, George TP. Collaborative testing in medical education: student perceptions and Long-Term knowledge retention. Med Sci EducJ Nurs Educ. 20202019;30(2):737–47. 10.1007/s40670-020-00944-x PMC8368897 34457732

[15] Rogers S, Gaffney TA, Caulfield E. Nursing student perception and performance with collaborative testing. J Nurs Educ Pract. 2021;11(5):54.

[16] Asal MGR, Atta MHR, El-Ashry AM, Hendy A, Kheder MEA, Mohamed AZ, El-Sayed AAI. International nursing students’ culture shock and academic engagement: The moderating role of resilience. Nurse Educ Today. 2024;145:106499. 10.1016/j.nedt.2024.106499. Epub ahead of print. PMID: 39577017. 10.1016/j.nedt.2024.106499 39577017

[17] Atta MHR, El-Sayed AAI, Alsenany SA, Hammad HA, Elzohairy NW, Asal MGR. Navigating transition shock: The role of system thinking in enhancing nursing process competency among early career nurses. Worldviews Evid Based Nurs. 2024 Nov 21. 10.1111/wvn.12757. Epub ahead of print. PMID: 39572034. https://doi.org/10.1111/wvn.12757. 10.1111/wvn.12757 39572034

[18] Vázquez-García M. Collaborative-group testing improves learning and knowledge retention of human physiology topics in second-year medical students. Adv Physiol Educ. 2018;42(2):232–9. 29616577 10.1152/advan.00113.2017

[19] Ali HFM, Mousa MAEG, Atta MHR, et al. Exploring the association between internet addiction and time management among undergraduate nursing students. BMC Nurs. 2024;23:632. 10.1186/s12912-024-02273-5. 39256720 10.1186/s12912-024-02273-5 PMC11389558

[20] Hussein Ramadan Atta MHR, Zoromba MA, Asal MGR, et al. Predictors of climate change literacy in the era of global boiling: a cross-sectional survey of Egyptian nursing students. BMC Nurs. 2024;23:676. 10.1186/s12912-024-02315-y. 39322950 10.1186/s12912-024-02315-y PMC11425957

[21] Amin SM, El-Sayed MM, El-Monshed AH. Hussein ramadan atta. The hidden link: dysmenorrhea, emotion regulation, and attitudes toward marriage in female nursing students. BMC Nurs. 2024;23:721. 10.1186/s12912-024-02341-w. 39379878 10.1186/s12912-024-02341-w PMC11463104

[22] Mohamed Ali Saleh N, Abd El-Naser Ali G, Gamal Mohamed M. Hassan Abd El Ftah S. Impact of critical thinking and problem solving skills on academic achievement among nursing students’. Egypt J Heal Care. 2021;12(2):932–45.

[23] Bovee B. The impact of collaborative testing on test anxiety.. 2016;4(1):1–23.

[24] Rushdan EE, Atta MHR, Nashwan AJ, Zoromba M, Ali HFM. Comparative analysis of engagement and academic self-concept among nursing students: differences in study modalities. Nurs Forum. 2024. 10.1155/2024/6621905.

[25] Hamed AEM, Hamza MF, El-Tawab NAA, Othman AA, Barakat AM, Atta MHR. Exploring the mediation role of hardiness in the relationship between feedback sensitivity and test anxiety among nursing students: a multi-site inquiry. BMC Nurs. 2025;24(1):421. 10.1186/s12912-025-02946-9. PMID: 40234885. 10.1186/s12912-025-02946-9 PMC11998217 40234885

[26] Mahoney JW, Harris-Reeves B. The effects of collaborative testing on higher order thinking: do the bright get brighter? Act Learn High Educ. 2019;20(1):25–37.

[27] Aydin S, Yerdelen S, Yalmanci SG, Göksu V. Academic motivation scale for learning biology: A scale development study. Egit Ve Bilim. 2014;39(176):425–35.

[28] Duane BT, Satre ME. Utilizing constructivism learning theory in collaborative testing as a creative strategy to promote essential nursing skills. Nurse Educ Today [Internet]. 2014;34(1):31–4. Available from: 10.1016/j.nedt.2013.03.005 10.1016/j.nedt.2013.03.005 23608232

[29] Kourmousi N, Xythali V, Theologitou M, Koutras V. Validity and reliability of the problem solving inventory (PSI) in a nationwide sample of Greek educators. Soc Sci. 2016;5(2).

[30] Utvær BKS, Haugan G. The academic motivation scale: dimensionality, reliability, and construct validity among vocational students. Nord J Vocat Educ Train. 2016;6(2):17–45.

[31] Valenzuela J, Nieto AM, Saiz C. Critical thinking motivational scale: A contribution to the study of the relationship between critical thinking and motivation. Electron J Res Educ Psychol. 2014;9(2):823–48.

[32] Efu SI. Exams as Learning Tools: A Comparison of Traditional and Collaborative Assessment in Higher Education. Coll Teach [Internet]. 2019;67(1):73–83. Available from: 10.1080/87567555.2018.1531282

[33] Rivaz M, Momennasab M, Shokrollahi P. Effect of collaborative testing on learning and retention of course content in nursing students. J Adv Med Educ Prof [Internet]. 2015;3(4):178–82. Available from: http://www.ncbi.nlm.nih.gov/pubmed/26457315%0Ahttp://www.pubmedcentral.nih.gov/articlerender.fcgi?artid=PMC4596384 PMC4596384 26457315

[34] Atta MHR, Rushdan EE, Elzohairy NW. The relationship between defensive pessimism, goal orientation, and self-esteem among nursing students. SAGE Open Nurs. 2024;10:23779608241293662. 10.1177/23779608241293662. 39525945 10.1177/23779608241293662 PMC11544683

[35] Radford M, Blount C. The Impact of Collaborative Testing on Teamwork and Collaboration in Nursing Students. 2023;2–4.

[36] Olvera-Lobo MD, Robinson BJ, Senso JA, Muñoz Martín R, Muñoz-Raya E, Murillo-Melero M, Quero-Gervilla E, Castro-Prieto MR, Conde-Ruano T. Student satisfaction with a web-based collaborative work platform. Perspectives: Stud Translatology. 2007;15(2):106–22. 10.2167/pst010.0.

[37] Connelly C. The Positive Influence of Collaborative Testing in the L2 Classroom. 2022;35–45.

[38] Levy D, Klinger M, Svoronos T, Levy D, Klinger M, Svoronos T. Two-Stage Examinations: Can Examinations Be More Formative Experiences? Faculty Research Working Paper Series. 2018;0–20.

[39] Amin SM, Demerdash DE, El-Sayed MM, et al. Navigating the fear: assessing nursing students’ concerns and preventive practices in response to Monkeypox in Egypt. BMC Nurs. 2025;24:23. 10.1186/s12912-024-02589-2. 39773708 10.1186/s12912-024-02589-2 PMC11708079

[40] Atta MHR, Madkour AMA, Hamad NIM, Abdallah HMM, Alkubati SA, Amin SM. Breaking barriers: the power of self-efficacy in combating occupational stigma and advancing gender equity in nursing education. Nurse Educ Today. 2025;106632. 10.1016/j.nedt.2025.106632. 10.1016/j.nedt.2025.106632 40022986

[41] Wiggs CM. Collaborative testing: Assessing teamwork and critical thinking behaviors in baccalaureate nursing students. Nurse Educ Today [Internet]. 2011;31(3):279–82. Available from: 10.1016/j.nedt.2010.10.027 10.1016/j.nedt.2010.10.027 21084136

[42] Jun Zhanga and QC. Collaborative learning in higher nursing education: A systematic review. J Prof Nurs. 2018;32(2):103–17. 10.1016/j.profnurs.2018.07.007 30243695

[43] Helmke BP. Improving Student Perceptions of Learning through Collaborative Testing. ASEE Annu Conf Expo Conf Proc. 2023.

[44] Atalla AD, Elamir H, Abou Zeid MA. Exploring the relationship between organisational silence and organisational learning in nurses: A cross-sectional study. J Nurs Adm Manag. 2022;30(3):702–15. 10.1111/jonm.13539. 10.1111/jonm.13539 35014104

