# Supplementary material for: Unlocking students’ potential beyond traditional exams: the influence of collaborative testing on nursing students’ retention and soft skills
Source: BMC Nurs. 2025 May 26;24:595. doi: 10.1186/s12912-025-03237-z (PMC12107850; doi:10.1186/s12912-025-03237-z)
Supplement: Supplementary file 1 — Supplementary Material 1 [file 12912_2025_3237_MOESM1_ESM.pdf]

Course Name: Emergency Nursing (022002218)
Academic Year 2023-2024 / Fall Semester
Final Written Exam

Date: Sunday 21st Jan 2024 **Time: 9:00 AM – 11:00 AM**

Student's Name: --Student's Code: -------------------

Exam Parts	Type of Questions	Allotted Score	Student's Score	Faculty's Signature
Part I	Multiple Choice	70		
Total		70		

Course Coordinators: Prof. Dr. Nagwa Reda - A. Prof. Amina H. Salem

Reviewing Committee **Date: 15/ 1/2024**

Prof. Nagwa Reda ……………………………..

A. Prof. Amina Hemida ……………………………..

Dr. Rawia Gamil ……………………………..

Multiple Choice Questions: **(70 Grades)**

Read the following questions and shade the correct answers in the bubble sheet.

1. **In conducting a primary survey on a trauma patient, which of the following measures is taken first?**
   a. Patient's allergy history.
   b. Check for responsiveness.
   c. Initiation of pulse oximetry.
   d. Complete set of vital signs.

2. **A 20-year-old male patient was brought to the emergency department (ED) with head trauma after a route traffic accident. Which of the following interventions has the highest priority?**
   a. Provide oxygen therapy.
   b. Initiate intravenous access.
   c. Do oral and nasal suctioning.
   d. Immobilize the cervical area.

3. **Regarding the patient in the above question (2), the patient became unresponsive and breathless. To open his airway, which of the following is the appropriate method?**
   a. Insertion of a nasopharyngeal airway.
   b. Insertion of an oropharyngeal airway.
   c. Using a jaw-thrust maneuver.
   d. Using head-tilt, and chin-lift maneuvers.

4. **Which of the following is a scoring system used to determine the severity of the trauma and the need to transport the victim to a trauma center?**
   a. AVPU score
   b. ABCDE score
   c. Glasgow coma score
   d. Revised trauma score

5. **A group of victims arrived at the scene of a terrorist attack. Which of the following injuries should be treated first?**
   a. Sprained ankle
   b. Sucking chest wound
   c. Abdominal evisceration
   d. Open fracture of the left forearm

6. **When the head is struck, causing the brain to move within the cranial vault and forcibly contact the opposite pole of the skull, this is considered as which of the following head injury mechanisms?**
   a. Rotational
   b. Acceleration
   c. Deceleration
   d. Coup–countercoup

7. **A patient admitted to the ED experienced Raccoon eyes. X–ray revealed that the patient had a basilar skull fracture. This means which of the following areas could be affected?**
   a. Left orbit
   b. Middle fossa
   c. Anterior fossa
   d. Posterior fossa

8. **Which of the following measures is the goal of treatment of patients admitted to ED with traumatic brain injury?**
   a. Avoid a seizure activity.
   b. Administer pharmacological thromboprophylaxis.
   c. Maintain a cerebral perfusion pressure of 30–60 mm Hg.
   d. Maintain tight glycemic control (blood glucose 150–250 mg/dl).
9. **A patient was admitted to the ED with a BP of 150/60 mm Hg and an intracranial pressure (ICP) of 20 mm Hg. Which of the following represents cerebral perfusion pressure (CPP)?**
   a. 52 mmHg
   b. 67 mmHg
   c. 70 mmHg
   d. 90 mmHg
10. **Which of the following assessment findings supports Cushing's triad in patients with increased intracranial pressure (ICP)?**
    a. BP 98/60 mmHg, pulse 55 beats/minute, temperature 37.2°C.
    b. BP 180/60 mmHg, pulse 55 beats/minute, respirations 10 breaths/minute.
    c. BP 120/80 mmHg, pulse 120 beats/minute, respirations 20 breaths/minute.
    d. BP 150/90 mmHg, pulse 99 beats/minute, respirations 24 breaths/minute.
11. **A patient is admitted to the ED with a head injury. His GCS is 7 so he is intubated and attached to a mechanical ventilator. When the nurse suctions the patient, which of the following interventions is important?**
    a. Suction the nasopharynx.
    b. Hyperoxygenate before, during, and after suctioning.
    c. Increase the tidal volume of the ventilator before suctioning.
    d. Turn the head from side to side to help reach the left bronchus.
12. **Which of the following is NOT TRUE about the different types of shock?**
    a. Septic shock is associated with cold clammy skin.
    b. Hypovolemic shock is associated with low cardiac output.
    c. Cardiogenic shock is associated with arrhythmia, MI, and myocarditis.
    d. Obstructive shock is associated with cardiac tamponade and pulmonary emboli.
13. **A patient is in the progressive stage of shock. If the shock is not corrected and tissue hypoxia occurs, what would happen to the patient's metabolism?**
    a. Nothing will occur.
    b. Vasomotor reflex reduces the pooling of blood in the microcirculation.
    c. Anaerobic metabolism, creating lactic acid and lowering the tissue pH.
    d. Metabolism would be unaffected by the lower oxygen level for 2-3 hours.
14. **A traumatic brain injury patient is experiencing bradycardia, hypotension, venous stasis in the extremities, and loss of temperature control. Which of the following types of shock is most evident?**
    a. Septic shock
    b. Cardiogenic shock
    c. Neurogenic shock
    d. Hypovolemic shock
15. **Which of the following is the CORRECT sequence of the renin-angiotensin-aldosterone system in the hypoperfusion state?**
    a. Angiotensin I → Angiotensin II → ACE → Renin → Angiotensinogen.
    b. Renin → Angiotensinogen → Angiotensin I → ACE → Angiotensin II.

c. Renin → Angiotensin I → Angiotensinogen → ACE → Angiotensin II.
d. Angiotensinogen → Renin → ACE → Angiotensin I → Angiotensin II.

16. A girl was admitted to the ED after being bitten by a snake. She experienced anaphylaxis. After establishing a patent airway, which of the following actions takes the highest priority?
a. IV administration of hydrocortisone.
b. Nebulized administration of Ventolin.
c. Parenteral administration of epinephrine.
d. Sublingual administration of nitroglycerin.

17. Which of the following represents the pathophysiological mechanisms of distributive shock?
a. Vasodilation.
b. Vasoconstriction.
c. Increase cardiac output.
d. Increase vascular resistance.

18. Which of the following conditions would create obstructive shock?
a. Fracture extremities
b. Tension pneumothorax
c. Acute abdominal bleeding
d. Intracranial hypertension

19. A patient's MRI imaging shows ischemia in the cerebellum a week after the patient suffered a stroke. What assessment finding correlates with this MRI finding?
a. Vision problems.
b. Language difficulty.
c. Balance impairment.
d. Impaired short-term memory.

20. Tissue plasminogen activator (tPA) is most effective in the treatment of stroke if it is administered at which of the following time frames?
a. 3 hours after the onset of stroke symptoms.
b. 6 hours after the onset of stroke symptoms.
c. 9 hours after the onset of stroke symptoms.
d. 12 hours after the onset of stroke symptoms

21. A nurse is educating a patient about transient ischemic attacks (TIAs). Which of the following is INCORRECT about this condition?
a. TIA requires immediate assessment and medical treatment.
b. TIA is a warning sign that an impending stroke may occur.
c. TIA is caused by a temporary decrease in blood flow to the brain.
d. TIA produces signs and symptoms that can last for several weeks.

22. Who of the following individuals is at the GREATEST risk of a stroke?
a. A teenager on oral contraceptives.
b. A patient in severe septic shock.
c. A person who has uncontrolled hypertension.
d. A person with well-controlled diabetes mellitus.

23. Which of these conditions does NOT predispose to ischemic stroke?
a. Sickle cell disease.
b. Atrial fibrillation.
c. Cerebral aneurysm.
d. Deep vein thrombosis.

24. When caring for a patient diagnosed with ischemic stroke, which of the following interventions is the priority when administering tissue plasminogen activator therapy (tPA)?

a. Educate the patient and family on stroke recovery.
b. Assess the patient's motor function to compare to baseline.
c. Assess the patient for recent history of bleeding or trauma.
d. Explain the purpose of tPA therapy to the patient and family.

25. A patient is admitted with uncontrolled atrial fibrillation. Which of the following types of strokes is this patient at MOST risk for?

a. Hemorrhagic.
b. Ischemic stenosis.
c. Ischemic embolism.
d. Ischemic thrombosis.

26. Which of the following body functions is regulated by the thyroid-producing hormones?

a. Digestion.
b. Temperature.
c. Metabolism.
d. Blood pressure.

27. As the nurse educating the patient about Grave's Disease, which of the following statements best describes this condition?

a. It is a complication of untreated hypothyroidism.
b. It is not associated with protruding eyes or a goiter.
c. It is caused by nodular goiters producing excessive amounts of T3 and T4.
d. It is caused by an autoimmune condition where antibodies act like TSH on the body.

28. Which of the following medication orders should a nurse question if ordered for a patient with thyroid storm?

a. Tapazole for a 30-year-old who is breastfed.
b. Aspirin is needed for a fever greater than 39 °C.
c. Inderal for a patient who reports having tachycardia.
d. Propylthiouracil "PTU" for a 25-year-old who is in the first trimester.

29. The physician orders a patient in thyroid storm to be started on Inderal. Which of the following patient's health history causes the nurse to question the doctor's order?

a. History of asthma.
b. History of cancer.
c. History of tachycardia.
d. History of mental illness.

30. Which of the following trends of vital signs characterizes thyroid storm?

a. Increased body temperature, decreased pulse, and increased blood pressure.
b. Increased body temperature, increased pulse, and increased blood pressure.
c. Increased body temperature, decreased pulse, and decreased blood pressure.
d. Increased body temperature, increased pulse, and decreased blood pressure.

31. A 35-year-old female with diabetic ketoacidosis is admitted to the hospital with the following profile: serum glucose 400 mg/dl, pH 7.12, K+ 4.4 mEq/L, and Na+ 141 mEq/L. Which of the following is the appropriate initial treatment?

a. Administration of normal saline and insulin.
b. Administration of normal saline, potassium, and insulin.
c. Administration of normal saline, potassium, insulin, and bicarbonates.
d. Administration of hypotonic sodium chloride (0.45%) solution, potassium, and insulin.

32. Which of the following side effects is possible for a patient taking anti-thyroid medications?
a. Eczema
b. Tachycardia
c. Agranulocytosis
d. Skin discoloration

33. In patients with Diabetic Ketoacidosis (DKA), which of the following respiratory patterns represents the respiratory system compensation for acidosis?
a. Bitot's
b. Cullen's
c. Trousseau's
d. Kussmaul's

34. Which of the following statements is INCORRECT about diabetic Ketoacidosis?
a. Blood glucose > 600 mg/dL
b. Ketones are present in the urine.
c. Metabolic acidosis is compensated by deep rapid breathing.
d. Potassium levels should be at least 3.3 or higher during treatment with insulin therapy.

35. Which of the following combinations represents the triad of diabetic ketoacidosis?
a. Hyperglycemia, ketosis & acidosis
b. Acidosis, polydipsia & polyphagia
c. Tachycardia, tachypnea & hyperglycemia
d. Hyperglycemia, hyperosmolarity, electrolyte imbalance

36. Which of the following pathogenesis wouldn't be seen in patients with non–ketotic hyperosmolar hyperglycemic state?
a. Acidosis
b. Dehydration
c. Hyperglycemia
d. Electrolytes Imbalance

37. Which of the following patients is most likely to develop diabetic ketoacidosis?
a. None of the options is correct.
b. A 35-year-old female newly diagnosed with Type 2 diabetes.
c. The patient did not eat the past 2 days and has been unable to take insulin.
d. The patient is newly diagnosed with Cushing's Disease and taking glucocorticoids.

38. A patient was admitted to the ED with GI bleeding, a history of Myocardial Infarction (MI), his INR was > 2, and received Warfarin. Which of the following treatments should be considered?
a. Triple therapy.
b. Fresh frozen plasma.
c. Platelets Transfusion.
d. Injection with adrenaline.

39. Which of the following treatments for peptic ulcer disease cannot be delivered via endoscopic therapy?
a. Triple therapy.
b. Mechanical clips.
c. Thermal hemostasis.
d. Injection with adrenaline.

40. If a patient has lower GI bleeding, which of the following would be the color of the blood?
a. Brown.
b. Green or yellow.
c. Black or tarry stools.
d. Bright red blood or maroon blood.

41. Which of the following interventions has the highest priority in a patient who comes to the ED vomiting blood and has a disturbed level of consciousness?
a. Insertion of ETT immediately.
b. Sending blood for cross-matching.
c. Insertion of two 18 gauge cannula.
d. Emergent endoscopy within 2 hours.

42. Triple therapy would be prescribed to treat a patient with GI bleeding by which of the following actions?
a. Depression of acid.
b. Eradication of H pylori.
c. Reversal of Anticoagulation.
d. Vasoconstriction of splanchnic blood flow.

43. A patient was admitted to the ED with severe hematemesis. He was hemodynamically unstable, and his laboratory results showed INR > 2 and a platelet count of 50.000. Which of the following interventions should be done immediately after securing the airway?
a. Endoscopy.
b. Transfusion of RBCs.
c. Reversal coagulation.
d. Insertion of Sengstaken.

44. Which of the following patients is more likely to develop pancreatitis?
a. Reinvent with renal problems and hypocalcemia.
b. A patient with a stone lodged in the pancreatic duct.
c. A 59-year-old male with a history of occasional alcohol use.
d. A client recovering from a myocardial infarction with hypercholesterolemia.

45. Which of the following actions of pancreatic enzymes can cause pancreatic damage?
a. Reflux into the pancreas.
b. Utilization by the intestine.
c. Autodigestion of the pancreas.
d. Clogging of the pancreatic duct.

46. Which of the following interventional measures have top priority in a patient with acute pancreatitis?
a. Nasogastric suction and parenteral nutrition.
b. Parenteral nutrition and effective pain control.
c. H2-receptor antagonist and parenteral octreotide.
d. Electrolyte resuscitation and effective pain control.

47. A patient with acute pancreatitis is reporting excessive thirst, excessive voiding, and blurred vision. As a nurse, which of the following is a priority?
a. Check the patient's blood glucose.
b. Provide a dark and calm environment.
c. Reassure the patient this is normal with pancreatitis.
d. Assist the patient with drinking a simple sugar drink like orange juice.

48. **A patient was admitted to the ED with acute pancreatitis and abdominal pain. Which of the following is a nonpharmacological pain relief action would be appropriate for this patient?**
    a. Encourage ambulation.
    b. Give frequent small sips of milk.
    c. Place a heating pad over the epigastrium.
    d. Have the patient lie in a lateral decubitus knee–bent position.
49. **Regarding the patient in the above question (48). The patient's pain became more severe and was indicated to administer narcotics. Meperidine (Demerol) 50 mg IM was prescribed instead of morphine because of it:**
    a. Is less addictive than morphine.
    b. Has fewer cognitive side effects.
    c. Has a faster onset of action than morphine.
    d. Causes fewer spasms of the sphincter of Oddi.
50. **Which patient below is at the <u>MOST</u> risk for developing autonomic dysreflexia?**
    a. A 24-year-old male patient with a traumatic brain injury.
    b. A 35-year-old male patient with a spinal cord injury below L6.
    c. A 15-year-old female patient with a spinal cord injury at C7.
    d. A 42-year-old male patient recovering from a hemorrhagic stroke.
51. **A patient with a spinal cord injury at level C8 is being cared for in the ED. Which of the following is the priority assessment?**
    a. Need for suctioning.
    b. Absent corneal reflex.
    c. Decerebrate posturing.
    d. Movement of only the right or left half of the body.
52. **Which of the following is a mechanism of secondary spinal cord injury (SCI)?**
    a. Release of catecholamines.
    b. Decrease of neurotransmitters.
    c. Hyperperfusion of the spinal cord.
    d. Immune cells leave the spinal cord.
53. **Which of the following types of incomplete spinal cord syndromes is caused by injury below lumber 2 and manifested by sciatica and bowel or bladder dysfunction?**
    a. Central cord syndrome.
    b. Cauda equina syndrome.
    c. Brown-Séquard syndrome.
    d. Posterior (Dorsal) cord syndrome.
54. **A patient with SCI complains of a sudden severe throbbing headache. Assessment of the patient reveals increased blood pressure (170/95) and bradycardia (48/minute), diaphoresis, and flushing of the face and neck. Which of the following is an appropriate action?**
    a. Adjust the temperature in the patient's room.
    b. Notify the physician about the change in status.
    c. Check the Foley tubing for kinks or obstruction.
    d. Administer the ordered acetaminophen (Tylenol).
55. **Which of the following combinations represents a tirade of tension pneumothorax?**
    a. Cardiac compromise, coma, acidosis.
    b. Collapsed lung, coagulopathy, acidosis.
    c. Collapsed lungs, mediastinal shift & tracheal deviation.
    d. Hypotension, mediastinal shift, and trachea in the midline.

56. **Which of the following is a sign that confirms the diagnosis of an open pneumothorax?**
    a. Bubbling around the wound
    b. Paradoxical chest movement
    c. Symmetrical chest movement
    d. Crepitus sound during palpation
57. **Which of the following is the appropriate location of the needle thoracentesis to remove tension pneumothorax?**
    a. Fifth intercostal space midaxillary line
    b. Fifth intercostal space midclavicular line
    c. Second intercostal space midaxillary line
    d. Second intercostal space midclavicular line
58. **Which of the following is the priority intervention for open pneumothorax?**
    a. Covering the wounds from all sides.
    b. Providing high-flow oxygen using a reservoir mask.
    c. Leaving the wounds uncovered, empty air entered the pleural space.
    d. Keeping the wound edges moist with gauze soaked with normal saline.
59. **Which of the following is the rationale for applying a sterile occlusive wound dressing on three sides in a patient with open pneumothorax?**
    a. To create a flutter valve.
    b. To fix the penetrating object.
    c. To recheck the wound each shift.
    d. To minimize skin inflammation due to plaster application
60. **Which of the following is included in Beck's triad in a patient with cardiac tamponade?**
    a. Low JVP, hypotension, and increased heart sound
    b. Low JVP, hypotension, and decreased heart sound
    c. High JVP, hypotension, and decreased heart sound
    d. High JVP, hypertension, and decreased heart sound
61. **What is one finding upon assessment that would indicate a suspected flail chest?**
    a. Increased stridor.
    b. Decreased O2 Sat.
    c. Difficulty breathing.
    d. Paradoxical breathing.
62. **During the assessment of a patient with penetrating abdominal wounds from a gunshot, a nurse notes bruising around the umbilicus. Which of the following terminology best describes this sign?**
    a. Cullen’s sign.
    b. Ballance’s sign.
    c. Chvostek’s sign.
    d. Grey Turner’’s sign.
63. **A young male patient has been brought to the emergency department with a knife wound to the abdomen with a protruded part of his intestine from the wound. Which of the following is the appropriate nurse response?**
    a. Apply pressure dressing to the wound.
    b. Cover the protruding viscera with saline-soaked, sterile gauze.
    c. Irrigate the protruding intestine with sterile water or normal saline.
    d. Don sterile gloves and attempt to push the organ back inside the wound.

64. Kehr's sign (left shoulder pain due to diaphragmatic irritation) indicates injury in which of the following abdominal organs?
a. Liver
b. Spleen
c. Stomach
d. Kidneys

65. Which of the following procedures is used to measure the intraabdominal pressure?
a. CT abdomen
b. MRI abdomen
c. Abdominal ultrasound
d. Foley's bladder catheter

66. Which of the following measurements indicates the diagnosis of abdominal compartment syndrome?
a. 5 mmHg
b. 7 mmHg
c. 12 mmHg
d. ≥20 mmHg

67. Non-surgical treatment of abdominal compartment syndrome is most likely involving which of the following measures?
a. Naso-gastric tube placement
b. Avoidance of muscle relaxants
c. Monitoring of intra-abdominal pressure once daily
d. Fluid resuscitation to a central venous pressure of 12 mm Hg

68. Which of the following blood vessel compressions would mostly contribute to decreasing cardiac output in patients with abdominal compartment syndrome?
a. Femoral vein
b. Abdominal aorta
c. Inferior vena cava
d. Superior vena cava

69. A patient who has pulmonary embolism may develop hypotension by which of the following mechanisms?
a. Pressure on the heart and reduced cardiac output.
b. Reduced blood delivery to the left side of the heart.
c. Reduced blood returns to the right side of the heart.
d. Reduced blood flow to the lung, which causes hypotension.

70. A patient with a massive pulmonary embolism will have an arterial blood gas analysis performed to determine the extent of hypoxia. Which of the following acid-base disorders is suspected?
a. Metabolic acidosis
b. Respiratory acidosis
c. Metabolic alkalosis
d. Respiratory alkalosis

END OF THE EXAM
GOOD LUCK
