# Supplementary material for: Unlocking students’ potential beyond traditional exams: the influence of collaborative testing on nursing students’ retention and soft skills
Source: BMC Nurs. 2025 May 26;24:595. doi: 10.1186/s12912-025-03237-z (PMC12107850; doi:10.1186/s12912-025-03237-z)
Supplement: Supplementary file 2 — Supplementary Material 2 [file 12912_2025_3237_MOESM2_ESM.pdf]

Course Name Emergency Nursing Course (022002218)
Academic Year 2023-2024 / First Semester
Midterm Written Exam

Date: Wednesday, November 22nd, 2023, **Time of Exam: 9 AM – 10 AM**

Student's Name: --Student's Code: -------------------

Exam Parts	Type of Question	Allotted Score	Student's Score	Faculty's Signature
Part I	Multiple Choice	40		
Total		40		

Course Coordinator: Prof. Dr. Nagwa Reda

Revised By:

Prof. Nagwa Reda ……………………………..

A. Prof. Amina Hemida ……………………………..

Dr. Rawia Gamil ………………………………

Multiple Choice Questions: (40 Grades)

Read the following questions carefully, choose the correct answer then shade it in the answer sheet.

1. **Which of the following is a center of the scope of emergency nursing?**
   a. A nurse
   b. **A patient**
   c. A physician
   d. An environment
2. **"A panic button" is a safety measure used to overcome which of the following risks in the emergency department?**
   a. Pain
   b. **Violence**
   c. Overcrowding
   d. Long waiting time
3. **"Vigilant" is one of the characteristics of an emergency nurse that means which of the following statements?**
   a. **Keep conscious/oriented**
   b. Understand cultural diversity
   c. Has cared and sympathetic behaviors
   d. Remain calm in stressful and high-pressure situations
4. **The system of 'triage' is based upon which of the following principles?**
   a. Treating first come first served
   b. Treating the quickest and easiest first
   c. Treating patients in order of priority
   d. Treating those that complaint the most first
5. **The ''yellow'' tag of the triage system is assigned to which of the following victims?**
   a. Has minor injuries and needs delayed care
   b. Has injuries that are so severe that death is imminent
   c. Cannot survive without immediate lifesaving interventions
   d. **Has potential serious injuries and needs care within 2-4 hours**
6. **The wounded victim is unable to walk, has a respiratory rate of 12, capillary refill is 8 seconds, and is unresponsive. The wounded victim is assigned to what tag color?**
   a. Green
   b. **Red**
   c. Yellow
   d. Black
7. **A group of victims arrived at the scene of a terrorist attack. Which of the following should receive the care first?**
   a. A 70–year–old woman with a pelvic fracture
   b. An 8–year–old girl with six superficial facial lacerations
   c. A 40–year–old man with respirations of 32 breaths/minute
   d. A 30–year–old man with no respirations or pulse for an hour

8. **Which of the following is <u>NOT</u> considered an immediate cause of death at the scene of the trauma?**
   a. Massive brain injury
   b. High spinal cord injury
   c. Rupture of liver or spleen
   d. Transection of the great vessels
9. **Which of the following represents the lethal triad of death associated with trauma?**
   a. Hyperglycemia, ketosis, and acidosis
   b. Alkalosis, hyperthermia, and bleeding
   c. Tachycardia, tachypnea, and hypotension
   d. **Hypothermia, acidosis, and coagulopathy**
10. **While resuscitating a trauma patient, which of the following is the right sequence of care?**
    a. Establish IV route, keep the airway open, immobilize C-spine, oxygenate
    b. Immobilize C-Spine, keep the airway open, oxygenate, and establish an IV route
    c. Oxygenate, immobilize the C-spine, keep the airway open, establish IV route
    d. Establish IV rout, keep the airway open, Oxygenate, and immobilize C-spine
11. **A victim arrives at the emergency department who suffered multiple injuries from a car accident. Which of the following assessments should take the highest priority?**
    a. Irregular pulse
    b. Unequal pupils
    c. A deviated trachea
    d. Ecchymosis in the flank area
12. **Which of the following ethical principles best describes the following situation? "The patient decides not to have an open-heart surgery despite coronary blockages".**
    a. Veracity
    b. Fidelity
    c. Autonomy
    d. Non-maleficence
13. **Which of the following ethical terms best describes "the prohibition of disclosure of patient's information gained in certain situations"?**
    a. Justice
    b. Veracity
    c. Maleficence
    d. Confidentiality
14. **Which of the following combinations of ethical principles has ethical and legal demands?**
    a. Justice & Veracity
    b. Fidelity & Justice
    c. Veracity & Maleficence
    d. **Beneficence & non – maleficence**
15. **Which of the following is not required for valid consent?**
    a. Voluntariness
    b. Being informed
    c. The ability to sign a consent form
    d. Having the capacity to make a decision

16. **Which of the following is a nurse's role in obtaining informed consent for implementing a specific medical or surgical procedure?**
    a. Explaining the procedure
    b. Performing the procedure
    c. **Witnessing the patient's approval**
    d. Providing psychological support

17. **Which of the following tasks could be delegated to the Unlicensed Assistive Personnel (UAP)?**
    a. Cleaning catheterization
    b. Formulating nursing diagnosis
    c. Formulating a nursing care plan
    d. Performing a physical assessment

18. **Which of the following conditions is considered a secondary traumatic brain injury?**
    a. Cerebral edema
    b. Brain concussion
    c. Basilar skull fracture
    d. Intracerebral hemorrhage

19. **When the head is struck, causing the brain to move within the cranial vault and forcibly contact the opposite pole of the skull, this is considered which of the following head injury mechanism?**
    a. Rotational
    b. Acceleration
    c. Deceleration
    d. Coup–countercoup

20. **Which of the following methods could be used to confirm that the leakage fluid is cerebrospinal fluid (CSF)?**
    a. Use a PH strip
    b. Send to lab for investigation
    c. Check for a halo sign on filter paper
    d. Check for a positive protein stick test

21. **A nurse is caring for a patient who sustained a head injury after falling 6 meters. She notes the presence of raccoon eyes., this finding suggests the presence of which of the following?**
    a. **Basal skull fracture**
    b. Congenital anomaly
    c. C1 spinal cord injury
    d. Intracerebral hemorrhage

22. **Which of the following is a goal of therapy during fluid resuscitation in traumatic brain injury patients?**
    a. A CVP of 15 mmHg
    b. A MAP of ≥70 mm Hg
    c. An UOP of 100 mL/hour
    d. A capillary refill of 8 seconds

23. **Which of the following is best described by Monroe Kellie's Hypothesis?**
    a. Cerebral perfusion
    b. Classification of brain injury
    c. Physiological electrical function of the brain cells
    d. Pressure - volume relationship within the intracranial cavity

24. **A patient is admitted to the ED with head trauma and is at risk for increased intracranial pressure. A nurse places a patient in a semi-flower position for which of the following reasons?**
    a. Promote adequate airway
    b. Prevent pulmonary congestion
    c. Facilitate venous return of blood from the brain
    d. Increase the circulating volume of blood to the brain
25. **A 23–year–old was admitted to ED with subarachnoid hemorrhage. Which of the following equations is used to calculate cerebral perfusion pressure?**
    a. Subtracts the radial pulse from the apical pulse
    b. Multiplies the stroke volume by the heart rate
    c. Divides the diastolic pressure plus the pulse pressure by 3
    d. Subtracts the intracranial pressure from the mean arterial pressure
26. **During an initial assessment of a patient with traumatic brain injury, Glasgow Coma Scale was 7. Which of the following actions has a top priority?**
    a. **Prepare for intubation**
    b. Perform tracheal suctioning
    c. Assess the patient's saturation
    d. Administer oxygen via nasal cannula
27. **Mr. A sustained a road traffic accident causing an intracerebral hemorrhage. Which of the following assessment findings requires immediate reporting and action?**
    a. SpO2 95%
    b. Potassium 5.2
    c. ICP 25 mm Hg
    d. Temperature 37 ^{o}C
28. **A patient develops increased intracranial pressure (ICP) and fever (40.8 C). The cooling blanket is used to decrease the ICP by which of the following mechanisms?**
    a. Decreasing the patient's metabolic rate
    b. Increasing the blood supply to the brain
    c. Increasing the oxygen supply to the lungs
    d. Bypassing the patient's owing temp-regulation mechanism
29. **A patient with a severe head injury attached to mechanical ventilation and his ICP is 22 mmHg. Hyperventilation was prescribed to perform which of the following actions?**
    a. Increases oxygen to the brain
    b. Dilates cerebral blood vessels
    c. Increases cerebral blood volume
    d. Promotes cerebral vasoconstriction
30. **A nurse is caring for a patient who sustained a head injury. He has an intracranial pressure monitor in place and his ICP is 35 mmHg. Which of the following medications would be prescribed?**
    a. Digoxin
    b. Mannitol
    c. Morphine
    d. Gentamycin

31. **Beck's triad associated with cardiac tamponade includes which of the following signs?**
   a. Raised JVP, hypotension, and increased heart sound
   b. Raised JVP, hypertension, and decreased heart sound
   c. Raised JVP, hypotension, and decreased heart sound.
   d. Minimum JVP, hypotension, and decreased heart sound
32. **Pericardiocentesis is an intervention used to treat which of the following conditions?**
   a. Cardiac tamponade
   b. Massive hemothorax
   c. Closed pneumothorax
   d. Tension pneumothorax
33. **Which of the following is the first priority intervention for the management of open pneumothorax?**
   a. Leave the wound uncovered
   b. Cover the wound from four sides
   c. Cover the wound from three sides
   d. Insert chest tube at the 5th intercostal space
34. **Which of the following are assessment findings of a victim with tension pneumothorax?**
   a. Bradypnea, hyper-resonance, & tracheal deviation to the unaffected side
   b. Bradypnea, hyper-resonance, & tracheal deviation to the affected side
   c. Tachypnea, hyper-resonance, & tracheal deviation to the affected side
   d. Tachypnea, hyper-resonance, & tracheal deviation to the unaffected side
35. **Which of the following is a top priority nursing diagnosis for patient with pulmonary embolism?**
   a. Risk for infection
   b. Risk for bleeding
   c. Impaired gas exchange
   d. Ineffective tissue perfusion
36. **Which of the risk factors contribute to hypercoagulability and development of DVT?**
   a. Immobility
   b. Malignancy
   c. Varicose veins
   d. Central venous catheterization
37. **A patient was admitted to the ED with severe hypoventilation and expected pulmonary embolism. The ventilation – perfusion scan denotes the hypoventilation to the dead space. This means which of the following?**
   a. The area of the lung being perfused but not ventilated
   b. The area of the lung not being perfused nor ventilated
   c. The area of the lung being ventilated but not perfused
   d. The area of the lung being well ventilated well perfused
38. **In autonomic dysreflexia, the nurse would report which of the following findings below the site of the spinal cord injury?**
   a. Absent reflexes
   b. Low blood pressure
   c. Flushed lower body

d. **Pale, cool lower extremities**

39. A patient with a spinal cord injury at level C3-4 is being cared for in the ED. Which of the following is the priority assessment?

a. **Monitor respiratory effort and oxygen saturation level**
b. Check blood pressure and pulse for signs of spinal shock
c. Assess the level at which the patient has retained mobility
d. Determine the level at which the patient has intact sensation

40. Which of the following pathological reflexes in a spinal cord injury patient is performed by flicking the nail of the middle finger and is associated with flexion of the ipsilateral thumb and/or index finger?

a. Halo sign
b. Babinski reflex
c. **Hoffmann's sign**
d. Bulbocavernosus reflex

END OF THE EXAM

GOOD LUCK
