# Supplementary material for: Unlocking students’ potential beyond traditional exams: the influence of collaborative testing on nursing students’ retention and soft skills
Source: BMC Nurs. 2025 May 26;24:595. doi: 10.1186/s12912-025-03237-z (PMC12107850; doi:10.1186/s12912-025-03237-z)
Supplement: Supplementary file 3 — Supplementary Material 3 [file 12912_2025_3237_MOESM3_ESM.pdf]

Department: Critical Care & Emergency Nursing Department

قسم: تمريض العناية الحرجة و الطوارئ

Course Name: Emergency Nursing (022002218)

Academic Year 2023-2024 / Fall Semester

Post-lecture Quiz (1)

Student's Name: _____________________ **ID:________________**

Read the following questions & write the letter of the best answer in the space provided.

________ **1. The wounded victim is unable to walk, has respiratory rate of 40, capillary refill is 6 seconds, and can't follow simple commands. The wounded victim is assigned what tag color?**

a. Green
b. Red
c. Yellow
d. Black

________ **2. In conducting a primary survey on a trauma patient, which of the following is considered as a priority for assessing airway stability?**

a. Initiation of pulse oximetry.
b. Complete set of vital signs.
c. Patient's allergy history.
d. Check for responsiveness

________ **3. When attending to a patient with head and neck trauma following a car accident, the nurse's initial action is to?**

a. Provide oxygen therapy.
b. Initiate intravenous access.
c. Immobilize the cervical area
d. Do oral and nasal suctioning.

________ **4. A patient is hospitalized with a flail chest. Which of the following signs best supports this condition?**

a. Inspiratory wheezes
b. Jugular vein distension
c. Paradoxical chest movement
d. Tracheal deviation to the affected side

________ **5. An elderly victim is hospitalized after falling and sustaining a pelvic fracture. A nurse monitors vital signs frequently to detect complications. Which of the following complications can result from an unstable pelvic fracture?**

a. Infection
b. Hypertension
c. Hypovolemic shock
d. Increased urine output

____

________ **6. A male patient has lost about 15% of his blood volume (about 750 mL) from a stab wound. The patient's vital signs are within normal limits. The medical team is likely to classify this hemorrhage as a….**

a. Class I
b. Class II
c. Class III
d. Class IV

________ **7. A group of patients arrives at the scene of a terrorist attack. Which of the following patients should receive the care first?**

a. A 70–year–old woman with a pelvic fracture
b. A 30–year–old man with no respirations or pulse
c. An 8–year–old girl with six superficial facial lacerations
d. A 40–year–old man with respirations of 32 breaths/minute

8. Which of the following combinations represents the lethal triad of death associated with trauma?

a. Hyperglycemia, ketosis, and acidosis
b. Alkalosis, hyperthermia, and bleeding
c. Hypothermia, acidosis, and coagulopathy
d. Tachycardia, tachypnea, and hypotension

________ **9. Which of the following priorities of triage encompasses patients who potentially have serious health problems but whose injuries are not immediately life-threatening?**

a. Priority 1
b. Priority 2
c. Priority 3
d. Priority 4

________ **10. Which of the following statements about triage is true?**

a. Triage is a detailed assessment that should lead to a clinical diagnosis
b. Triage should be undertaken as soon after arrival in the ED as possible
c. Triage only ever needs to be undertaken once in a patient's ED journey
d. Triage is a low-risk activity and can be safely undertaken by junior staff with little training

End of the Quiz

Good Luck & Best Wishes

Course Coordinator
