# Supplementary material for: Unlocking students’ potential beyond traditional exams: the influence of collaborative testing on nursing students’ retention and soft skills
Source: BMC Nurs. 2025 May 26;24:595. doi: 10.1186/s12912-025-03237-z (PMC12107850; doi:10.1186/s12912-025-03237-z)
Supplement: Supplementary file 4 — Supplementary Material 4 [file 12912_2025_3237_MOESM4_ESM.pdf]

Department: Critical Care & Emergency Nursing Department

قسم: تمريض العناية الحرجة و الطوارئ

ALEXANDRIA UNIVERSITY

Course Name: Emergency Nursing (022002218)

Academic Year 2023-2024 / Fall Semester

Post-lecture Quiz (2)

Student's Name: ____________________ ID: ________________

Read the following questions & write the letter of the best answer in the space provided.

________ **1. A patient is being cared for after a traumatic brain injury. During an initial assessment, the nurse performs the Glasgow Coma Scale and gives the patient a score of 8. Which of the following responses is appropriate to manage the patient's respiratory rate?**

a. Prepare for intubation
b. Administer oxygen via nasal cannula
c. Administer oxygen via a non-rebreather mask
d. Remove oxygen and assess the patient's pulse oximetry

________ **2. The nurse suspects a basilar skull fracture in which of the following areas if there is ecchymosis behind the left ear (Battle's sign)?**

a. The left orbit
b. The base of the anterior fossa
c. The base of the middle fossa
d. The base of the posterior fossa

________ **3. Which of the following is best described by Monroe Kellie's Hypothesis?**

a. Cerebral perfusion
b. Classification of injury
c. Physiological electrical function of the brain cells
d. Pressure - volume relationship within the intracranial cavity

________ **4. A patient is admitted to the ED with head trauma and at risk of increased intracranial pressure. The nurse places a patient in a fowler's position with the head of the bed 30 degrees to:**

a. Promote adequate airway
b. Prevent pulmonary congestion
c. Facilitate venous return of blood from the brain
d. Increase the circulating volume of blood to the brain

________ **5. A 23–year–old was admitted to ED with a hemorrhagic stroke, he has been unconscious since admission. An intracranial pressure sensor was inserted. To calculate cerebral perfusion pressure, the nurse:**

a. Subtracts the radial pulse from the apical pulse
b. Multiplies the stroke volume by the heart rate
c. Divides the diastolic pressure plus the pulse pressure by 3
d. Subtracts the intracranial pressure from the mean arterial pressure

________ 6. **A patient with a severe head injury attached to mechanical ventilation and his ICP is 22 mmHg. Hyperventilation was prescribed to perform which of the following actions?**

a. Increase oxygen to the brain
b. Dilate cerebral blood vessels
c. Increase cerebral blood volume
d. Promote cerebral vasoconstriction

________ 7. **A patient with a head–closed injury has an ICP of 14 mmHg, which of the following statements best interprets the pressure reading?**

a. Within acceptable range
b. Too low, possibly initiating herniation
c. Too high, needs immediate interventions
d. Low, indicating medical intervention is successful

8. **A 35 – old – year patient diagnosed with bacterial meningitis is at risk for increasing ICP. Which action is appropriate?**

a. Keep the head of the bed flat
b. Avoid hyperoxygenation before suctioning
c. Avoid activities that stimulate the Valsalva maneuver
d. Encourage hyperextension of the neck and extremities

________ 9. **After a car crash, a patient is hospitalized with head trauma. A nurse is monitoring the cerebral perfusion pressure. Which of the following parameters must be monitored closely?**

a. Pupils
b. Respirations
c. Systole & Diastole
d. Speech patterns

________ 10. **Which of the following interventions is contraindicated for a patient who is admitted with a suspected basilar skull fracture in the anterior fossa?**

a. Endotracheal intubation
b. Placement of an oral airway
c. Insertion of a nasogastric tube
d. Insertion of an indwelling urinary catheter

End of the Quiz

Good Luck & Best Wishes

Course Coordinator
