# Supplementary material for: Unlocking students’ potential beyond traditional exams: the influence of collaborative testing on nursing students’ retention and soft skills
Source: BMC Nurs. 2025 May 26;24:595. doi: 10.1186/s12912-025-03237-z (PMC12107850; doi:10.1186/s12912-025-03237-z)
Supplement: Supplementary file 5 — Supplementary Material 5 [file 12912_2025_3237_MOESM5_ESM.pdf]

Department: Critical Care & Emergency Nursing Department

قسم: تمريض العناية الحرجة و الطوارئ

ALEXANDRIA UNIVERSITY

Course Name: Emergency Nursing (022002218)

Academic Year 2023-2024 / Fall Semester

Post-lecture Quiz (3)

Student's Name: ______________________ ID: _________________

Read the following questions & write the letter of the best answer in the space provided.

________ 1. **A young male patient has been brought to the emergency department with a knife wound to the abdomen with a protruded part of his intestine from the wound. Which of the following is the appropriate nurse response?**
   - **a.** Apply a pressure dressing to the wound.
   - **b.** Cover the protruding viscera with saline-soaked, sterile gauze.
   - **c.** Irrigate the protruding intestine with sterile water or normal saline.
   - **d.** Don sterile gloves and attempt to push the organ back inside the wound

________ 2. **Splenic injury most likely results from blunt abdominal trauma. Therefore, it is most appropriate for practitioners to ask about recent trauma when a patient presents with which of the following symptoms?**
   - **a.** Low back pain
   - **b.** Central abdominal pain
   - **c.** Left upper quadrant pain
   - **d.** Right lower shoulder pain

________ 3. **Which of the following statements is TRUE about the blunt injuries to the abdomen?**
   - **a.** May cause peritonitis
   - **b.** May cause intestinal obstruction
   - **c.** Rarely need an urgent laparotomy
   - **d.** May cause gastroduodenal ulceration

________ 4. **Non-surgical treatment of abdominal compartment syndrome is most likely involving which of the following measures?**
   - **a.** **Naso-gastric tube placement.**
   - **b.** Avoidance of muscle relaxants.
   - **c.** Fluid resuscitation to a central venous pressure of 12 mm Hg.
   - **d.** Monitoring of intra-abdominal pressure once daily.

________ 5. **Which of the following is not included in the triad of symptoms associated with splenic rupture?**
   - **a.** **Belly Button**
   - **b.** Pleural effusion
   - **c.** Left lower lobe atelectasis
   - **d.** Elevation of the left hemidiaphragm

________ 6. **Which of the following is a LATE sign of the development of a tension pneumothorax?**
   - **a.** Dyspnea
   - **b.** Hypotension
   - **c.** Tachycardia
   - **d.** Tracheal deviation

________ 7. **Which of the following is the appropriate location of the needle thoracentesis to remove tension pneumothorax?**
   - **a.** Fifth intercostal space midaxillary line
   - **b.** Fifth intercostal space midclavicular line
   - **c.** Second intercostal space midaxillary line
   - **d.** Second intercostal space midclavicular line

8. **Which of the following is the priority intervention for open pneumothorax?**
   - **a.** Covering the wounds from all sides
   - **b.** Providing high-flow oxygen using a reservoir mask
   - **c.** Leaving the wounds uncovered to empty air enter the pleural space
   - **d.** Keeping the wound edges moist with gauze soaked with normal saline

________ 9. **Which of the following is the rationale for applying a sterile occlusive wound dressing on three sides in a patient with open pneumothorax?**
   - **a.** To create a flutter valve
   - **b.** To fix the penetrating object
   - **c.** To recheck the wound each shift
   - **d.** To minimize skin inflammation due to plaster application

________ 10. **Which of the following signs does the nurse expect to find in a patient with a flail chest?**
   - **a.** Symmetrical chest movement
   - **b.** Flail segment elevation during inhalation
   - **c.** Mediastinal shift with tracheal deviation
   - **d.** Flail segment depression during inhalation

End of the Quiz

Good Luck & Best Wishes

Course Coordinator
