# Supplementary material for: Unlocking students’ potential beyond traditional exams: the influence of collaborative testing on nursing students’ retention and soft skills
Source: BMC Nurs. 2025 May 26;24:595. doi: 10.1186/s12912-025-03237-z (PMC12107850; doi:10.1186/s12912-025-03237-z)
Supplement: Supplementary file 6 — Supplementary Material 6 [file 12912_2025_3237_MOESM6_ESM.pdf]

Department: Critical Care & Emergency Nursing Department

قسم: تمريض العناية الحرجة و الطوارئ

Course Name: Emergency Nursing (022002218)

Academic Year 2023-2024 / Fall Semester

Post-lecture Quiz (4)

Student's Name: ____________________ **ID: ________________**

Read the following questions & write the letter of the best answer in the space provided.

Mr. X was admitted to the EMD following a road traffic accident (RTA). His CT revealed SCI at a level of T 12. Currently, his vital signs include BP: 90/60 mmHg, pulse: 55 beats/minute, temperature 36.2°C. Physical examination revealed: warm flushed skin, absent bladder and rectal sphincter, and priapism.

Use the above scenario to answer questions 1 - 3

________ 1. **Which of the following best describes the condition that Mr. X developed?**
   - **a.** Spinal Shock
   - **b.** Neurogenic shock
   - **c.** Autonomic dysreflexia
   - **d.** Brown-Sequard Syndrome

________ 2. **After Mr. X received 1000 mL of normal saline 0.9%, his CVP is 10 mmHg, but B.P is still 82/40 mmHg. The nurse will anticipate the administration of which of the following?**
   - **a.** Morphine
   - **b.** Nitroglycerine
   - **c.** Phenylephrine
   - **d.** Norepinephrine

________ 3. **Which of the following is indicating that the fluid resuscitation is effective for Mr. X?**
   - **a.** Urine output increase from 5mL/hr. to 25mL/hr.
   - **b.** Core body temperature increase from 36.8°C to 37.1°C
   - **c.** Pulse pressure decreased from 35 mm Hg to 28 mm Hg
   - **d.** Respiratory rate increase from 22 breaths/min to 28 breaths/min

________ 4. **A patient with a spinal cord injury (SCI) complains about a severe throbbing headache that suddenly started a short time ago. Assessment of the patient reveals increased blood pressure (168/94) and decreased heart rate (48/minute), diaphoresis, and flushing of the face and neck. What action should you take first?**
   - **a.** Adjust the temperature in the patient's room.
   - **b.** Notify the physician about the change in status.
   - **c.** Check the Foley tubing for kinks or obstruction.
   - **d.** Administer the ordered acetaminophen (Tylenol).

________ 5. **A patient with a spinal cord injury at level C3-4 is being cared for in the ED. What is the priority assessment?**
   - **a.** Monitor respiratory effort and oxygen saturation level
   - **b.** Check blood pressure and pulse for signs of spinal shock.
   - **c.** Assess the level at which the patient has retained mobility.
   - **d.** Determine the level at which the patient has intact sensation.

---

6. **Which of the following patients is at the highest risk for ARDs?**
   - **a.** A 75–year–old patient with a massive MI
   - **b.** A 50–year–old patient with an atrial Fibrillation
   - **c.** A 22–year–old female taking oral contraceptives
   - **d.** A 38–year–old patient with alcoholism and acute pancreatitis

____

7. **A nurse is teaching a patient's family about ARDS, which of the following statements best describes ARDS?**
   - **a.** Inability of the cardiopulmonary system to efficiently remove CO2
   - **b.** A form of noncardiogenic pulmonary edema
   - **c.** Lung disease in which alveolar lose their elasticity, causing collapse
   - **d.** Decreased cardiac output, causing fluid back up into the alveoli

____

8. **How does the prone position affect the lungs of patients with acute respiratory distress syndrome (ARDS)**
   - **a.** It helps the patient relax
   - **b.** It decreases pulmonary vascular resistance
   - **c.** It reduces the need for mechanical ventilation
   - **d.** It restores ventilation to the dorsal areas of the lung

____

9. **To manage a mechanically ventilated patient with ARDS, which of the following would best decrease intrapulmonary shunting?**
   - **a.** Increasing the PEEP
   - **b.** Increasing the tidal volume
   - **c.** Increasing the expiratory time
   - **d.** Increasing the respiratory rate

____

10. **A nurse is assessing a female patient with multiple traumas who is at risk for developing acute respiratory distress syndrome. The nurse assesses for which earliest signs of acute respiratory distress syndrome.**
    - **a.** Too Low PaO2, Bilateral wheezing, Bradypnea
    - **b.** High PaO2 Inspiratory crackles, Bilateral wheezing
    - **c.** Low PaO2, Intercostal retractions, Bradypnea
    - **d.** Low PaO2, Dyspnea, Tachypnea

End of the Quiz

Good Luck & Best Wishes

Course Coordinator
