# Supplementary material for: Unlocking students’ potential beyond traditional exams: the influence of collaborative testing on nursing students’ retention and soft skills
Source: BMC Nurs. 2025 May 26;24:595. doi: 10.1186/s12912-025-03237-z (PMC12107850; doi:10.1186/s12912-025-03237-z)
Supplement: Supplementary file 7 — Supplementary Material 7 [file 12912_2025_3237_MOESM7_ESM.pdf]

Department: Critical Care & Emergency Nursing Department

قسم: تمريض العناية الحرجة و الطوارئ

Course Name: Emergency Nursing (022002218)

Academic Year 2023-2024 / Fall Semester

Post-lecture Quiz (5)

Student's Name: ____________________ **ID: ________________**

<u>Read the following questions & write the letter of the best answer in the space provided.</u>

________ **1. Which of the following is <u>NOT TRUE</u> about the different types of shock?**
- **a.** Hypovolemic shock is associated with low cardiac output
- **b.** Septic shock is associated with cold clammy skin
- **c.** Cardiogenic shock is associated with arrhythmia, MI, and myocarditis
- **d.** Obstructive shock is associated with cardiac tamponade, pneumothorax, and pulmonary emboli

________ **2. A patient is in the progressive stage of shock. If the shock is not corrected and tissue hypoxia occurs, what would happen to the patient's metabolism?**
- **a.** Anaerobic metabolism, creating lactic acid and lowering the tissue pH
- **b.** Vasomotor reflex reduces pooling of blood in the microcirculation
- **c.** Metabolism would be unaffected by the lower oxygen level for the 2-3 hours
- **d.** Nothing will occur

________ **3. Which of the following types of shock requires administration of <u>Epinephrine</u> in addition to standard treatments?**
- **a.** Septic
- **b.** <u>Anaphylactic</u>
- **c.** Cardiogenic
- **d.** Hemorrhagic

________ **4. Which of the following stages of shock is associated with the worsening of tissue hypoperfusion and the onset of ischemia and metabolic imbalances, including acidosis?**
- **a.** Initial phase
- **b.** Developing phase
- **c.** Progressive stage
- **d.** Irreversible stage

________ **5. Which of the following represents the pathophysiological mechanisms of distributive shock?**
- **a.** Vasodilation
- **b.** Vasoconstriction
- **c.** Increase cardiac output
- **d.** Increase vascular resistance

________ **6. Whose of the following individuals is at the GREATEST risk of a stroke?**
- **a.** A teenager on oral contraceptives
- **b.** A patient in severe septic shock

c. A patient with well-controlled diabetes mellitus

d. A patient who has uncontrolled hypertension

________ **7. Tissue plasminogen activator (tPA) is a drug used to treat stroke. Which of the following statements is INCORRECT regarding tPA?**

a. Bleeding is a side effect of this drug

b. It may also be used in hemorrhagic stroke

c. It should be given within a certain time frame

d. Cannot be given with heparin

8. Which patient below is at the most risk for a hemorrhagic stroke?

a. A 55-year-old with atrial flutter

b. A 65-year-old with carotid stenosis

c. An 89-year-old with atherosclerosis

d. An 88-year-old with a history of brain aneurysm

________ **9. Which of the following patients is a candidate for tissue plasminogen activator (tPA) for the treatment of stroke?**

a. CT is positive for hemorrhage.

b. A patient whose blood pressure is 220/110

c. A patient who received Heparin 24 hours ago

d. A patient who is showing an ischemic stroke

________ **10. Which of the following cerebral arteries has the highest tendency for occlusion?**

a. Carotid

b. Middle

c. Anterior

d. Posterior

End of the Quiz

Good Luck & Best Wishes

Course Coordinator
