# Supplementary material for: Unlocking students’ potential beyond traditional exams: the influence of collaborative testing on nursing students’ retention and soft skills
Source: BMC Nurs. 2025 May 26;24:595. doi: 10.1186/s12912-025-03237-z (PMC12107850; doi:10.1186/s12912-025-03237-z)
Supplement: Supplementary file 8 — Supplementary Material 8 [file 12912_2025_3237_MOESM8_ESM.docx]

**Students’ Satisfaction with Collaborative Testing Survey**

**Dear Student**

Thank you for taking part in the study. I kindly request you to spare a few minutes to complete the survey. As a member of the student group who participated in 5 post-lecture collaborative tests, please use a 5-point Likert scale to rate your satisfaction, where 5 means strongly agree, 4 means agree, 3 means neutral, 2 means disagree, and 1 means strongly disagree. Additionally, please indicate whether you would recommend this assessment style for use in other courses.

| **#** | **Items** | **Strongly Agree (5)** | **Agree (4)** | **Neutral (3)** | **Disagree (2)** | **Strongly Disagree (1)** |
| --- | --- | --- | --- | --- | --- | --- |
| **CRITICAL THINKING & DECISION MAKING** | | | | | | |
| **The collaborative test** | | | | | | |
|  | Helped me to how to reason correctly. |  |  |  |  |  |
|  | Enhanced my understanding and ability to synthesize and integrate the course content. |  |  |  |  |  |
|  | Motivated me to use more thought processes. |  |  |  |  |  |
|  | Provided me with the opportunity to discuss incorrect answers and fill in knowledge gaps, therefore improving understanding of the material. |  |  |  |  |  |
|  | Worked well with colleagues to analyze and reach a final consensus answer to quiz questions. |  |  |  |  |  |
|  | Helped me to express opinions, discuss, debate, negotiate, and ask questions. |  |  |  |  |  |
|  | Helped students solve problems, make decisions, plan, and organize their work. |  |  |  |  |  |
| **COMMUNICATION & TEAMWORK** | | | | | | |
| **The collaborative test** | | | | | | |
|  | Provided me with a more positive relationship between students and faculty. |  |  |  |  |  |
|  | Improved my level of involvement during the discussions. |  |  |  |  |  |
|  | Allowed every member of my group to contribute to the weekly discussions. |  |  |  |  |  |
|  | Enhanced peer communication and teaching because it allowed me to achieve a better understanding of the material as well. |  |  |  |  |  |
|  | Enabled the students to work collaboratively. |  |  |  |  |  |
|  | Helped me to communicate professionally. |  |  |  |  |  |
|  | Enabled me to work in a group and to be more successful than when I worked alone. |  |  |  |  |  |
| **SELF-CONFIDENCE & SELF-ESTEEM & TEST ANXIETY** | | | | | | |
| **The collaborative Test** | | | | | | |
|  | Improved my self-esteem and provided me with more positive relationships with my colleagues. |  |  |  |  |  |
|  | Improved my self–confidence because I achieved a better understanding of the material as well. |  |  |  |  |  |
|  | Reduced my test anxiety. |  |  |  |  |  |
| **ACADEMIC MOTIVATION & ACCOUNTABILTY & ENGAGEMENT** | | | | | | |
| **The collaborative test** | | | | |  |  |
|  | Motivated me to be more successful than when working alone. |  |  |  |  |  |
|  | Helped me develop increased accountability. |  |  |  |  |  |
|  | Increased my motivation to attend the lecture weekly. |  |  |  |  |  |
|  | Improved my positive behaviors and decreased negative behaviors toward the educational process as a whole. |  |  |  |  |  |
| **OVERALL SATISFACTION** | | | | | | |
| **The collaborative test** | | | | | | |
|  | It was effective & I would recommend this assessment method for other courses. |  |  |  |  |  |

**End of the Survey**

**Thank you**
